# New Compound Sets Identified from High Throughput Phenotypic Screening Against Three Kinetoplastid Parasites: An Open Resource

**DOI:** 10.1038/srep08771

**Published:** 2015-03-05

**Authors:** Imanol Peña, M. Pilar Manzano, Juan Cantizani, Albane Kessler, Julio Alonso-Padilla, Ana I. Bardera, Emilio Alvarez, Gonzalo Colmenarejo, Ignacio Cotillo, Irene Roquero, Francisco de Dios-Anton, Vanessa Barroso, Ana Rodriguez, David W. Gray, Miguel Navarro, Vinod Kumar, Alexander Sherstnev, David H. Drewry, James R. Brown, Jose M. Fiandor, J. Julio Martin

**Affiliations:** 1 Molecular Discovery Research, Tres Cantos Medicines Development Campus, GlaxoSmithKline, Tres Cantos, Spain; 2 Diseases of the Developing World (DDW), Tres Cantos Medicines Development Campus, GlaxoSmithKline, Tres Cantos, Spain; 3 Department of Microbiology, Division of Parasitology, New York University School of Medicine, New York, NY, USA; 4 Drug Discovery Unit, Division of Biological Chemistry and Drug Discovery, University of Dundee, Dundee, UK; 5 Instituto de Parasitología y Biomedicina “López-Neyra” Consejo Superior de Investigaciones Cientificas, Granada, Spain; 6 Computational Biology, Quantitative Sciences, GlaxoSmithKline, Collegeville, PA, USA; 7 Computational Biology, Quantitative Sciences, GlaxoSmithKline, Medicines Research Center, Stevenage, Hertfordshire, UK; 8 Chemical Sciences, Molecular Discovery Research, GlaxoSmithKline, Research Triangle Park, NC, USA

## Abstract

Using whole-cell phenotypic assays, the GlaxoSmithKline high-throughput screening (HTS) diversity set of 1.8 million compounds was screened against the three kinetoplastids most relevant to human disease, i.e. *Leishmania donovani*, *Trypanosoma cruzi* and *Trypanosoma brucei*. Secondary confirmatory and orthogonal intracellular anti-parasiticidal assays were conducted, and the potential for non-specific cytotoxicity determined. Hit compounds were chemically clustered and triaged for desirable physicochemical properties. The hypothetical biological target space covered by these diversity sets was investigated through bioinformatics methodologies. Consequently, three anti-kinetoplastid chemical boxes of ~200 compounds each were assembled. Functional analyses of these compounds suggest a wide array of potential modes of action against kinetoplastid kinases, proteases and cytochromes as well as potential host–pathogen targets. This is the first published parallel high throughput screening of a pharma compound collection against kinetoplastids. The compound sets are provided as an open resource for future lead discovery programs, and to address important research questions.

Half a billion people are at risk of contracting diseases caused by kinetoplastid parasites of the genera *Leishmania* and *Trypanosoma*^1^. This group of flagellated protozoans causes African trypanosomiasis (*T. brucei*), Chagas disease (*T. cruzi*) and various clinical manifestations of leishmaniasis (*Leishmania* spp.). In particular, *L. donovani* and *L. infantum* are responsible for life-threatening visceral leishmaniasis. An estimated 20 million individuals are infected with kinetoplastid pathogens, resulting in extensive suffering and around 95,000 deaths per year^1^. Despite this considerable disease burden, effective drug treatments for these parasites are lacking or inadequate and new therapies are required^2^,^3^.

Although these protozoans are transmitted by different insects and the human diseases they cause are clinically distinctive, much of their molecular and cellular biology is similar^1^. They are defined by the presence of a DNA-containing region, the ‘kinetoplast', in their single large mitochondrion; have similar genomic organization and cellular structures (e.g. a single flagellum for motion, and glycosomes); and undergo morphological changes during their life cycles in the insect and vertebrate hosts. The genome of each parasite exceeds 8,000 genes, more than 6,000 being common orthologs^4^. Analysis of the genomes of *L. major, T. cruzi* and *T. brucei* (known as TriTryp) has revealed many common core metabolic functions as well as pathways that might reflect specific adaptations to environments within their insect and vertebrate hosts^4^. Strikingly, approximately 50% of these genomes encode for hypothetical proteins that do not resemble orthologs in the human genome^1^. In addition, several human protein classes are not represented in kinetoplastid genomes, e.g. no orthologs have been found for tyrosine kinases^5^. These differences suggest that there may be essential proteins that can be exploited as selective targets for chemotherapy.

This paper reports the application of whole-cell phenotypic assays against *L. donovani*, *T. cruzi* and *T. brucei* to screen the GlaxoSmithKline HTS diversity set of 1.8 million compounds. This is the first parallel HTS program which has been disclosed for any pharma compound set against the three kinetoplastids most relevant to human disease. Three kinetoplastid chemical boxes have been assembled and all data are publically available to encourage research and drug discovery efforts in combating these devastating infections.

## Results

### High throughput screening (HTS) campaigns and hit identification

The 1.8 million GlaxoSmithKline HTS screening collection was tested against *L. donovani*, *T. cruzi* and *T. brucei* between October 2012-May 2014 using a primary whole-cell phenotypic screen as described in the Methods. All compounds were tested at a final assay concentration of 5 μM in the *L. donovani* and *T. cruzi* assays and at 4.2 μM in the *T. brucei* assays. Primary hits were identified using algorithms developed in-house^6^. For each of the three phenotypic (primary) screens, one corresponding orthogonal assay was conducted to prove genuine activity and help to rule out false activity caused by assay interference. HTS campaigns are described in Supplementary Table 1, HTS results are summarized in Supplementary Figure 1 and the HTS progression cascade in Supplementary Figure 2.

#### Leishmania donovani

Growth inhibition of free-living *L. donovani* amastigotes in axenic cultures was determined in an assay adapted from de Ryker, *et al*.^7^. At an average statistical cut-off of >30%, 67,400 primary hits were identified; an overall hit rate of 4%. Using the same assay, confirmatory activity above the cut-off in at least one replicate was displayed for 32,200 compounds. These remaining compounds were tested in an intracellular assay of *L. donovani*-infected THP1-derived macrophages^8^. The cut-off obtained using the amastigotes per macrophage output was >34% and the corresponding hit confirmation rate was 31%, resulting in ca. 5,500 active compounds selective for *L. donovani* over macrophage cells. Using physicochemical parameters^9^, such as molecular weight >500 Da, calculated Property Forecast Index (cPFI) < 8, and <5 aromatic rings, the number of hits was reduced to 4,700. Compound potency (pIC_50_) was determined in a dose–response experiment and acute cytotoxicity of the compounds was assessed using the HepG2 assay (see Methods). Consequently, 351 non-cytotoxic anti-*L. donovani* compounds were identified.

#### Trypanosoma cruzi

Growth inhibition was determined using NIH-3T3 fibroblasts infected with a recombinant *T. cruzi* strain expressing beta-galactosidase as an intracellular reporter, as adapted from Bettiol, *et al.*^10^. The average statistical cutoff was >27% inhibition and the obtained overall hit rate was 7.7%, i.e. 130,678 active compounds. This list was reduced to 45,100 compounds by increasing the cut-off for biological response to >45%, excluding compounds with historical annotation in GSK databases for cytotoxicity *versus* the HepG2 cell line (pIC_50_ > 6), and including only those compounds with sub-μM IC_50_ values, cPFI < 8, and aromatic rings < 4. Duplicate confirmation experiments were performed plus an interference assay against the host cell (i.e. NIH-3T3 fibroblasts). Based on >70% inhibition in the *T. cruzi* assay and <25% in the NIH-3T3 interference assay, 3,985 compounds were selected for dose–response and tested in parallel in the primary *T. cruzi* assay, the 3T3 host cell interference assay and for cytotoxicity against HepG2. A total of 2,310 compounds were identified with a *T. cruzi* pIC_50_ > 5 and a selectivity index > 10. These compounds were tested in an intracellular imaging assay in H9c2 cells (rat cardiomyoctes)^11^. Also, because sterol 14α-demethylase (CYP51) inhibitors have known activity against *T. cruzi*^12^, hits were triaged based on *T. cruzi* CYP51 inhibition data obtained using a recently developed assay^13^. Compounds without CYP51 activity or a selectivity index > 10 for *T. cruzi versus* CYP51 were first selected. CYP51 inhibitors with a lower index were selected only if highly potent against *T. cruzi* (pIC_50_ > 6). Overall, these investigations identified 500 non-cytotoxic anti-*T. cruzi* compounds.

#### Trypanosoma brucei

A resazurin fluorescent *T. brucei* whole-cell viability assay was used, derived from Sykes & Avery^14^. An average cut-off value of >40% resulted in 27,600 hits; a 1.5% overall hit rate. Confirmatory screening identified 15,200 compounds displaying a response above cut-off in at least one duplicate. A total of 4,200 compounds were selected for dose–response studies based on >80% growth inhibition in the confirmation step, cPFI < 8, aromatic rings < 5, molecular weight < 500 Da, and *in silico* determination of potential CNS penetration^15^. As well as the primary assay, these compounds were screened against *T. brucei* using an orthogonal ATP-based luminescence assay^16^, and acute cytotoxicity was evaluated against HepG2. Based on a pIC_50_ > 6 and selectivity > 10, there were 700 non-cytotoxic anti-*T. brucei* compounds identified.

### Anti-kinetoplastid chemical boxes

**Figure 1:**
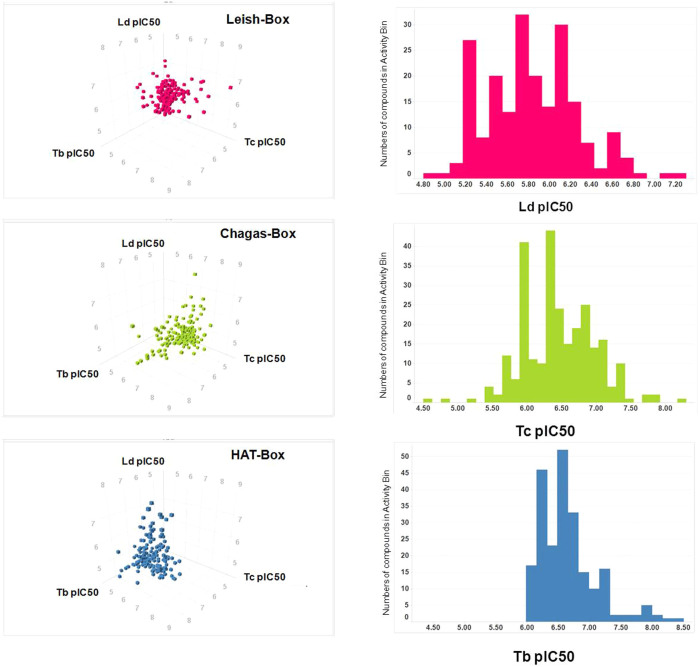
Distribution of potency by chemical box (*Leishmania donovani* in pink, *Trypanosoma brucei* in blue and *T. cruzi* in green) and parasite. Left panels: 3D scatter plot of pIC_50_ values for each compound against *L. donovani* (Ld pIC_50_, as inhibition of amastigotes per macrophage readout from imaging assay), *T. cruzi* (Tc pIC_50_, as inhibition of amastigotes per cell readout from imaging assay) and *T. brucei* (Tb pIC_50_, as inhibition of FLINT from fluorescent assay). Right panels: Distribution of potency for all compounds of each of the three chemical boxes (~200 compounds distributed into 40 potency bins).

Selection of representative chemical boxes for the three kinetoplastids started from the most potent, specific, and non-cytotoxic compounds in the dose–response outputs of each screen after having filtered for lead-like properties as described in the Methods. In order to generate representative boxes with high chemical diversity and potency, compounds were clustered initially by similarity using a complete-linkage algorithm^17^, and a threshold of 0.55. Secondly, they were sorted by decreasing potency (i.e. pIC_50_). Compounds were then selected so that all the clusters were represented in the final ranked boxes by no more than two members with the highest potency. The final boxes contained 592 compound entries; 192 were active against *L. donovani* (Leish-Box), 222 against *T. cruzi* (Chagas-Box) and 192 against *T. brucei* (HAT-Box) (Figure 1, Supplementary Table 2 and available at http://ebi.ac.uk/chemblntd). The three anti-kinetoplastid chemical boxes showed little overlap, pointing to specific mechanisms of growth inhibition or structural divergence across molecular targets in each parasite. Three compounds were in both the Leish-Box and Chagas-Box, nine in both the Chagas-Box and HAT-Box and one compound was present in all three chemical boxes.

### Biological profiling and chemical characterization

Database searches in ChEMBL showed that 88% of compounds in the three chemical boxes were not previously published. The literature annotation for the remaining 12% of compounds was unrelated to activity against *Leishmania* or *Trypanosoma*. Furthermore, these kinetoplastid chemical boxes do not contain analogs (Tanimoto coefficient > 0.7) to drugs currently used in the clinic for leishmaniasis, Chagas disease or sleeping sickness. Thus, they represent highly novel chemical diversity for potential starting points in drug discovery for kinetoplastid anti-parasitic agents. Only five compounds were natural products; a similar proportion to the screening collection.

There was no correlation between compound potency as determined by the axenic *versus* intracellular *L. donovani* assays (Supplementary Figure 3a). One explanation is that, in the intracellular assay, the compound must pass through two host cell membranes (i.e. plasma membrane and phagolysosome) plus the parasite cell membrane^7^, whereas in the axenic assay only the parasite membrane must be crossed. Also, there are significant pH changes from the extracellular media (acidic in the axenic assay, neutral in the intra-macrophage assay) to cytosol (neutral) to the parasitophorous vacuole (acidic pH 5.4). A reduced metabolism within the host cell may also impair drug activity against intra-macrophage *L. donovani* amastigotes, and these are slow-replicating, compared to axenic amastigotes^7^. In contrast, for *T. cruzi* and *T. brucei* there was good correlation for compound potency between the primary and orthogonal assays (Supplementary Figure 3b, 3c). This is as expected because the biological system for the assay differs only in the readout (i.e. fluorescent reporter *versus* imaging in *T. cruzi* and fluorescent-resazurin *versus* luminescent-ATP in *T. brucei*) or the host cell (i.e. NIH-3T3 fibroblasts *versus* H9c2 cardiomyocytes in *T. cruzi*).

As mentioned previously, the preclinical and clinical pipeline for Chagas disease is already well populated with CYP51 inhibitors^12^. In the Chagas-box, 42% of compounds represented a sub-set that was clearly differentiated from the overall compound population, exhibiting a pIC_50_ > 6 in both biochemical CYP51 and cellular anti-*T. cruzi* imaging assays (Supplementary Figure 4). These compounds may be flagged as putative CYP51-targeting hits. Conversely, 44% of compounds were CYP51-inactive; the remaining 14% poorly active.

The two imaging assays employed to determine compound activity against intracellular *L. donovani* and *T. cruzi* allowed simultaneous measurement of the number of amastigotes and infected cells as a dual assay readout. Interestingly, compounds in the Leish-Box and Chagas-Box showed good correlations between both anti-parasitic readouts, indicating parasite eradication (Supplementary Figure 5).

The screening methods and selection processes produced relatively small shifts in the distributions of clogP and molecular weight towards higher values, in many cases not significant (Supplementary Figure 6a, b) (Student t-test for means, data not shown). For the number of aromatic rings, the distributions were centered at 3 rings; none of the compounds had >4 aromatic rings (Supplementary Figure 6c). As expected from the guided triage process, most compounds lie in appropriate lead-like space^18^.

## Discussion: modes of action hypotheses

**Figure 2:**
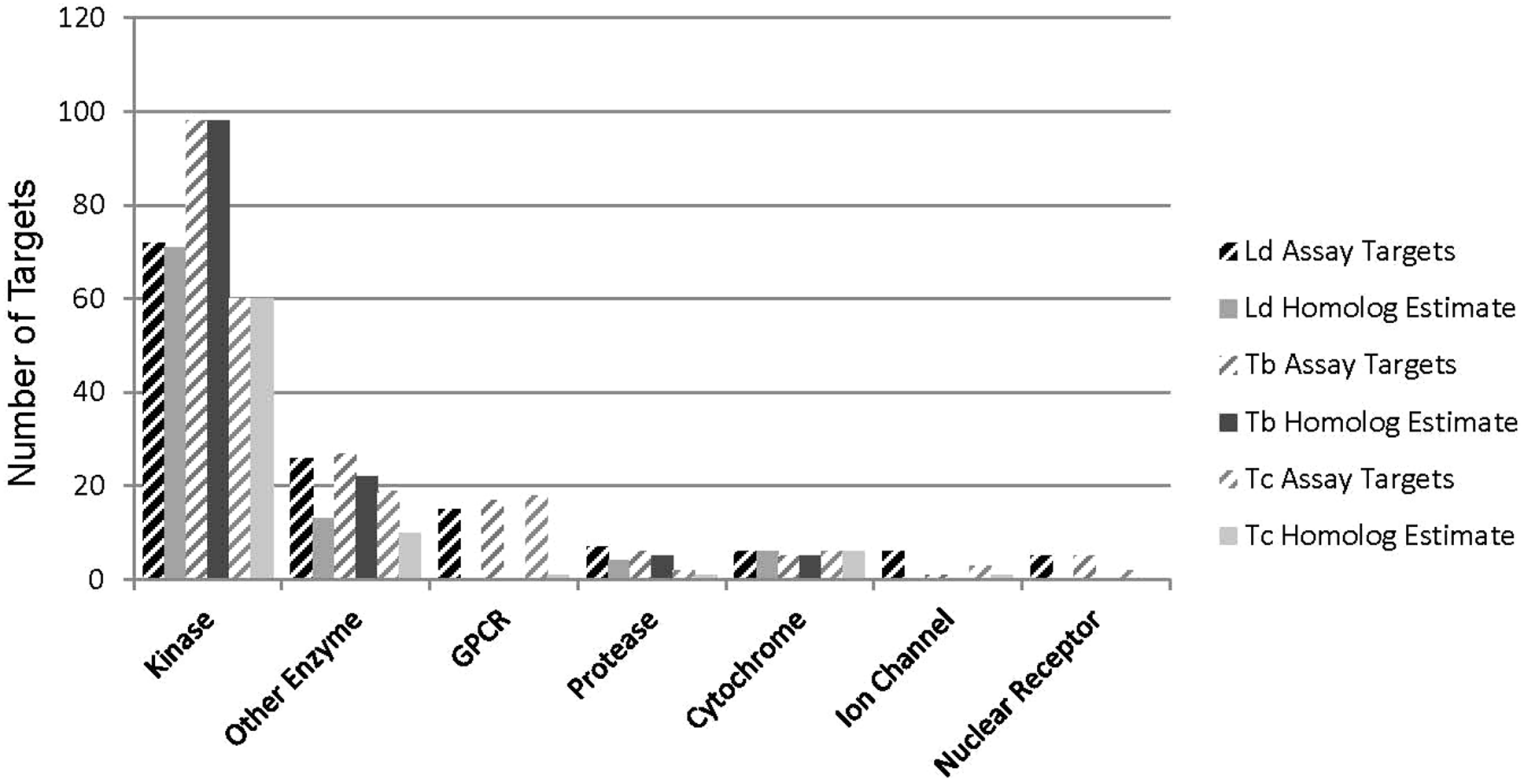
Histogram of assignment of positive compounds based on historical assay data and homology of human protein targets to kinetoplastid genomes. Histograms are clustered by assays for *Leishmania donovani* (Ld), *Trypanosoma brucei* (Tb) and *T. cruzi* (Tc). The threshold above which compound efficacy against specific human targets was considered significant was defined as pIC_50_ ≥ 5 for inhibition or antagonist assays, and pEC_50_ ≥ 5 for agonist, activation or modulator assays (i.e. overall pXC_50_ ≥ 5). Kinetoplastid homologs to human proteins were assigned based on BLASTP E-values ≤ 1.0e–05. Some compounds met the criteria for multiple human proteins (cross-hatched bars) while some kinetoplastid proteins had homology to multiple human proteins (solid bars).

To assist target identification efforts, we used historical GSK screening data to generate modes of action hypotheses for the compounds in the three kinetoplastid chemical boxes. Based on conservative thresholds (i.e. overall pXC_50_ ≥ 5.0, see Methods) for agonist or antagonist assays, compounds were associated with human protein targets which were used as BLASTP queries against the respective parasite genome^19^. The number of compounds meeting target specificity criteria was similar across *L. donovani* (80 compounds) and *T. brucei* (82 compounds) but lower for *T. cruzi* (46 compounds) (Figure 2).

‘Kinase' was the most frequent target class (Table 1). A range of human kinase inhibitor chemotypes were present in the three boxes, including common hinge binding motifs such as pyrimidines, pyrrolopyrimidines, azaindoles, and benzimidazoles. Kinase inhibitors are a potentially important novel class of anti-kinetoplastid therapeutics because of high chemotype diversity in pharmaceutical compound collections and their potency in disrupting parasite survival^20^. Compared to the human genome which encodes over 450 kinases^21^, the kinomes of *Leishmania* and *Trypanosoma* sp. are less diverse, having <200 protein kinases^5^. Kinome-wide RNAi screens of *T. brucei* suggest that at least 43 kinases have essential roles in cell cycle and differentiation by *in vitro* culture analysis^22^, and others might prove to be essential only when tested *in vivo*. Several putative *T. brucei* kinase targets have had cell death or arrest phenotypes reported in kinome-wide RNAi knock-down experiments^22^. Although kinome-wide RNAi studies have not been published for *L. donovani* and *T. cruzi*, kinase inhibitors have activity against *L. donovani in vitro*^23^. The present compound set might be interacting with 34–40 serine/threonine kinases, depending upon the parasite.

**Figure 3:**
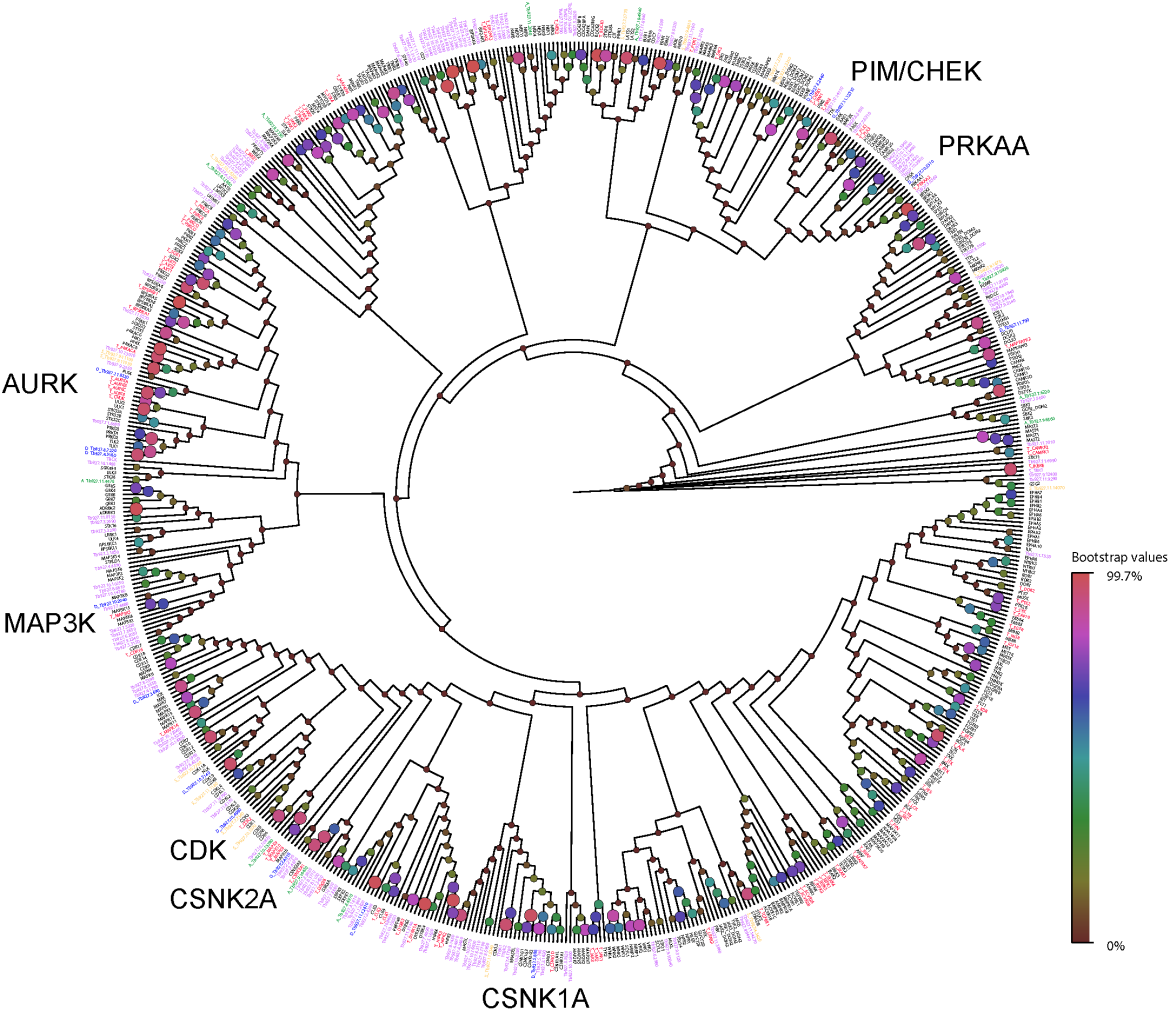
Neighbor-joining phylogenetic tree of combined human and *Trypanosoma brucei* str. 927 kinomes. Human kinase targets with putative compounds are colored and prefixed (T_red); all human kinases are colored black. *T. brucei* kinases are colored and prefixed for the RNAi phenotypes death (D_blue), arrest (A_dark green) or slow (S_orange) according to Table 1 in Jones *et al.*^22^. All other *T. brucei* kinases are colored violet. A few key clusters of human target and essential *T. brucei* kinases are labeled. The size and color of circles on the nodes represent support in 1000 bootstrap replicates.

A phylogenetic tree of the entire complement of human and *T. brucei* protein kinases was constructed (Figure 3), with additional annotations for those kinases with reported growth defects^22^. Several *T. brucei* kinases with cell death or arrest phenotypes cluster with human kinases which also met our historical assay specificity criteria, including aurora kinases (AURK), CDK, CSNK1/2, MAP3K, PIM/CHEK, and PRKAA. Some kinases are potential drug targets, such as AURK which are known to be involved in cell cycle progression, exist as single copy genes in kinetoplastids^24^ and have small molecule inhibitors with *in vitro* activity against *T. brucei*, and *L. major*^25^. The phylogenetic tree maps inhibitors of related human kinases against a single kinetoplastid isoform, illustrating the ancestral relationship of *T. brucei* kinases to multiple human homologues. These human–kinetoplastid kinase clusters provide starting points for further modes of action validation, with the caveat that many kinase inhibitors inhibit multiple, distantly related enzymes^26^. In addition to serine/threonine inhibitors kinases, several lipid kinases (phosphatidylinositol-kinases) were identified (Table 1). Lipid kinases also have a much lower complement in kinetoplastids compared to humans^27^, and could be important targets for either direct-acting anti-parasite drugs or disrupters of host–pathogen interactions^28^.

Potential modes of action hypotheses for the chemical box compounds were suggested against other kinetoplastid proteins, mainly phosphosdiesterases (PDEs), cysteine peptidases and cytochromes (CYPs) (Table 2). All these proteins have been previously proposed as new kinetoplastid drug targets and some are under active investigation. Phosphodiesterase C is conserved across *Leishmania* sp. and *Trypanosoma* sp. where it is essential for disposal of cyclic nucleotides^29^. Several PDE inhibitors have been tested against kinetoplastids, in particular *T. cruzi*^30^ and *T. brucei*^31^. Kinetoplastid cysteine peptidases are homologous to mammalian cathepsins B and L peptidases and perform essential roles. *T. brucei* requires active cysteine protease to deactivate the protective action of human apolipoprotein LI^32^,^33^. Kinetoplastid cytochromes are also a growing area of new drug development. CYP51 inhibition clears Chagas disease in mouse models^34^, and ferric reductase, which has cytochrome P450 sequence homology, is the primary mechanism of host iron uptake by *T. brucei*^35^. Several recent papers highlight the large diversity of structures that can bind to CYP51^36^,^37^,^38^, suggesting that a wide range of heterocyclic moieties can serve as a key pharmacophore binding to the cytochrome metal group. The large number of potential metal-binding pharmacophores in the kinetoplastid chemical boxes is striking, i.e. 39 meta-substituted pyridyl compounds, 20 *para*-substituted pyridyl compounds, and 20 imidazole-containing compounds. There are also an unusually high number of methylene dioxy containing compounds (28 of them). Although we could find no record of this moiety being a key feature of parasite CYP51 inhibitors, and indeed none of these were found to be *T. cruzi* CYP51 inhibitors, it does interact with human cytochrome P450s. Although it is unlikely that all compounds with these potential metal interacting moieties are working through a parasite cytochrome, it is an area that should be explored in more depth.

Another functional group that appears at an unusually high rate (62 compounds) is the nitro-substituted aryl group, including nitro-pyrazoles, nitro-triazoles, nitro-furans, nitro-thiophenes, and nitrobenzenes. This is perhaps expected as nifurtimox and benznidazole are nitro-substituted aromatics used clinically for Chagas disease. These two drugs are substrates for a parasite type I nitroreductase and are converted into metabolites toxic to the parasite^39^. There is no mammalian homologue to this target, suggesting a potentially useful therapeutic window. There has been increased interest in nitro drugs to treat these parasitic diseases^40^, and the wide range of nitro-aromatic compounds present in these sets facilitates further substrate selectivity studies.

**Figure 4:**
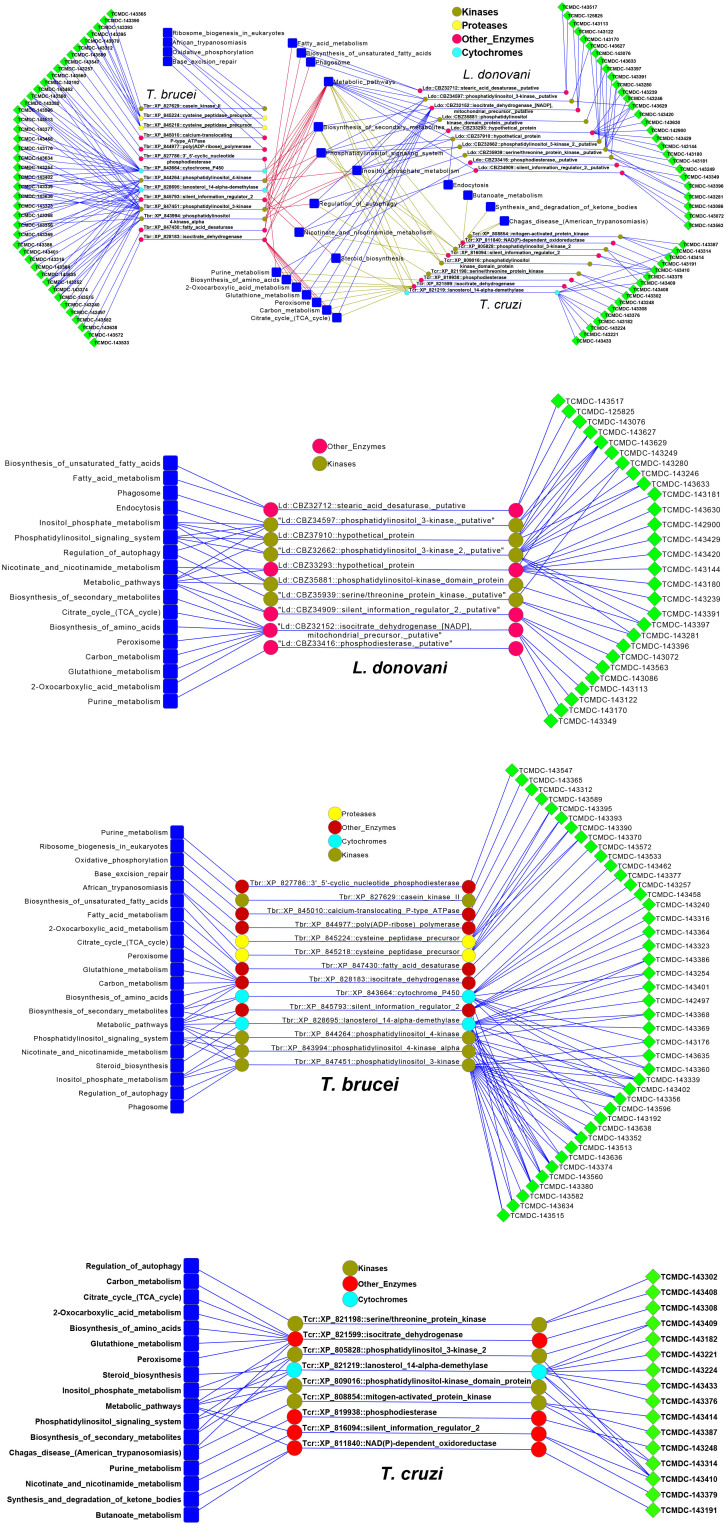
Pathway network analysis of combined and individual kinetoplastid target hypotheses. Pathways were assigned using KEGG and visualized using Cytoscape^42^. Proteins of three species are grouped and colored according to general functional groups (kinases, proteases, other enzymes and cytochromes).

For those compounds putatively associated with protein targets, we developed a preliminary systems biology network view of targeted pathways based on all known KEGG pathways for the kinetoplastid species^41^,^42^. The analysis included compounds with both human and kinetoplastid putative protein targets, i.e. 31, 48 and 62 compounds were analyzed for *T. cruzi*, *T. brucei* and *L. donovani*, respectively. These compounds are associated to 33 proteins in these parasites. Description of these proteins was taken from the NCBI Gene database (http://www.ncbi.nlm.nih.gov/gene). Pathway interactions were mapped for the individual kinetoplastid species and as a combined three species map (Figure 4).

Some additional predictions of homologous protein targets were included in this map, for which there were fewer compounds. A key caveat for these analyses is that the kinetoplastid KEGG reference pathways are incomplete^41^. For example, there are only 91 known pathways for *T. brucei*, *versus* 288 for humans. Thus, not all kinetoplastid proteins were associated with pathways. However, the total network still included 42 compounds for *T. brucei* (68% of all compounds analyzed), 16 (52%) for *T. cruzi*, and 28 (58%) for *L. donovani*. Other compounds target proteins with no known pathways. Although there were some species-specific pathways for *T. brucei* (n = 4), *T. cruzi* (n = 3) and *L. donovani* (n = 1), the majority of hypothesized targets (and compounds) could be assigned pathways common to all three parasites, including ‘biosynthesis of secondary metabolites', ‘metabolic pathways', ‘phosphatidylinositol signaling system (and metabolism)', ‘regulation of autophagy', ‘nicotinate and nicotinamide metabolism' and ‘steroid biosynthesis'. Interestingly, there were some proteins, e.g. silent information regulator 2 (XP 816094, XP 845793 and CBZ34909 for *T. cruzi, T. brucei* and *L. donovani*, respectively), with the same biological description and pathway in all three species. The compounds targeting these proteins may, therefore, disrupt this pathway in the same manner.

There were also several compounds associated with human proteins with no known homologs in kinetoplastids, such as G-coupled protein receptors (GPCRs), nuclear receptors, ion channels and transporters. These compounds potentially act on evolutionarily unrelated targets or essential proteins that, although structurally and functionally similar to the human targets, have no significant primary amino acid homology. Importantly, two assays in this study used parasites cultured in human host cells (*L. donovani* in macrophages and *T. cruzi* infecting fibroblasts). Thus, modulation of host–pathogen interactions could be an indirect mode of action for some compounds (with either homologous or non-homologous human targets). For example, *L. donovani* potentially modulates the cAMP signaling pathways mediated by the GPCR EP2 or PTGER2^43^. Disruptors of host–parasite interactions could lead to novel anti-kinetoplastid therapeutic strategies, less susceptible to the selection pressures which drive drug resistance in the parasite.

This paper reports for the first time the outcome from a parallel high throughput screening of a pharma compound collection (1.8 million compounds) against the three most relevant *Leishmania* and *Trypanosoma* parasites causative of human disease. Three corresponding kinetoplastid chemical boxes were identified, encompassing a wide variety of potential targets, such as kinases, proteases, and cytochromes as well as potential host-pathogen interaction targets. The outcomes of this research are publically disclosed in full, and physical samples of the three kinetoplastid chemical boxes are available to collaborators upon request. We expect this information will enable the scientific community to address relevant research questions and seed lead discovery programs that eventually deliver innovative treatments for these important, but neglected diseases.

## Methods

### Parasites and mammalian cell cultures

LLC-MK2 cells (green monkey kidney epithelial cells) were purchased from the European Cell Cultures Collection (ECACC reference 85062804) and used to expand the *T. cruzi* parasite population. NIH-3T3 cells (mouse endothelial fibroblasts) were made available by GSK-Biological Reagents and Assay Development Department (BRAD, Stevenage, UK). H9c2 (rat cardiomyocytes) were purchased at the European Cell Cultures Collection (ECACC, Salisbury, UK). *T. cruzi* parasites from the Tulahuen strain expressing β-galactosidase were kindly provided by Dr. Buckner (University of Washington, Seattle, USA; Buckner et al., 1996). Parasites were maintained in culture by weekly infection of LLC-MK2 cells. Trypomastigote forms were obtained from the supernatants of LLC-MK2 infected cultures harvested between days 5 and 9 of infection, as described elsewhere^10^. THP-1 cells (human monocytic leukemia) were made available by GSK-Biological Reagents and Assay Development Department (BRAD, Stevenage, UK). LdBOB axenic amastigotes expressing eGFP were kindly provided by Manu de Rycker from Dundee University. *Trypanosoma brucei brucei* bloodstream form Lister 427, was provided by Miguel Navarro from Instituto Lopez Neyra (Consejo Superior de Investigaciones Cientificas, Granada, Spain). HepG2 (human hepatoma) was provided by GSK-Biological Reagents and Assay Development Department (BRAD, Stevenage, UK).

#### Leishmania donovani

Primary growth inhibition assay: Assay plates (1536-well) were prepared by adding 30 nL per well of compound. For single shot assay, the final compound concentration was 5 μM. For potency determinations, eleven-point one in three dilution curves were generated with a top concentration of 50 μM. The assay was adapted from de Rycker *et al.*^7^. Briefly, eGFP LdBOB axenic amastigotes were harvested, fixed and counted in a CASY cell counter (Roche-Applied Science). A final concentration of 1.5 × 10^5^ cells per well were prepared in amastigote media containing 500 U/mL penicillin/streptomycin (Invitrogen™) and 6 μL was dispensed to each experimental well using a Multidrop Combi dispenser (Thermo Scientific). 6 μL of assay media was dispensed to control columns used as reference for 100% compound response. Following incubation for 72 h at 37°C, 5% CO_2_, 2 μL of resazurin was added to each well at a final concentration of 0.5 mM in phosphate buffered saline (PBS) with IGEPAL (Sigma-Aldrich), 0.05% (v/v). The plates were incubated for 4 h at room temperature and resorufin fluorescence was detected using a PerkinElmer EnVision plate reader with excitation at 528 nm and emission at 590 nm.

Intra-macrophage *L. donovani* assay: Assay plates (384-well) were prepared by adding 250 nL of compound to each well. For single shot assay, the final compound concentration was 5 μM. For potency determinations, eleven-point one in three dilution curves were generated with a top concentration of 50 μM. The assay was adapted from de Rycker *et al.*^7^. Briefly, THP-1 cells were grown previously in CELLMASTER roller bottles (Greiner cat. # 680048) at a concentration of 9 × 10^5^ cells/mL for 72 h. The cells were monitored using an optical microscope and counted using a CASY cell counter. Then they were differentiated in 225 cm^3^ T-FLASK in the presence of 30 nM of PMA (phorbol 12-myristate 13-acetate) (Sigma-Aldrich) at a final concentration of 6 × 10^5^ cells/mL. Following incubation for 24 h at 37°C, 5% CO_2_, cell differentiation were visually confirmed using an optical microscope. Cells were washed twice using cell culture media. Cells were infected using eGFP LdBOB axenic amastigotes at a multiplicity of infection of 10 (i.e. 6 × 10^6^ parasites/mL) in the T-FLASK containing differentiated THP-1 cells. Each T-FLASK was incubated overnight and the remaining parasite was removed washing three times with sterile DPBS. The infected cells was harvested by treatment with 0.05% (w/v) trypsin plus 0.48 mM EDTA for 5 min and an aliquot was fixed and counted in a CASY cell counter. A cell preparation with a final concentration of 1.6 × 10^5^ cells/mL was prepared in assay media consisting of RPMI (Invitrogen™), 2% FBS (Gibco) and 25 mM sodium bicabonate (Invitrogen™) containing 30 nM of PMA. Infected cells were plated onto assay plates containing compounds (3000 cells/well, 50 μL) using a Multidrop Combi dispenser. Amphotericin B (SigmaAldrich) was used as positive control of 100% compound response and was prepared by diluting the compound in an aliquot of infected cells for a final concentration of 2 μM. Treated cells were dispensed into the control column using a Multidrop Combi dispenser. Plates were incubated for 96 h at 37°C, 5% CO_2_ and then fixed with 4% (v/v) formaldehyde-PBS for 30 min at room temperature. After fixation, the wells were washed twice with 100 μL PBS using an EL406 multi well platewasher (BioTek), stained with 10 μg/mL DAPI in PBS plus 0.1% (v/v) Triton X-100 for 30 min at room temperature, and washed twice with 50 μL PBS. Finally, 50 μL of PBS was added to each well and the plates were sealed and read on a high-content microscope (Opera QEHS) using a 20x objective, 3 fields per well. Two exposure images were taken for each well using 405 nm and 488 nm laser excitation. Automated image analysis was performed with a script developed on Acapella® High Content Imaging and Analysis Software (PerkinElmer). THP-1 cell count, average number of amastigotes per macrophage and percent of infected cells were reported for each well.

#### Trypanosoma cruzi

Primary growth inhibition assay: The assay developed was adapted from the one previously described by Bettiol, *et al.*^10^. Active compounds in this assay format will target intracellular *T. cruzi* amastigotes growing in NIH-3T3 murine fibroblasts, though to a smaller extent they may also target free swimming trypomastigotes and/or the host–parasite interactions required for the parasite invasion. The assay was performed in tissue culture surface treated 1536 well plates. This assay was run at single shot at 5 μM compound concentration per well. The biological reagents consisted of NIH-3T3 host cells and trypomastigotes parasitic cells; both cell types were set to a 3.3 × 10^5^ cells/mL dilution in assay DMEM by means of a CASY cell counter device using a 60 μm capillary. 6 μL of the mixture were dispensed per well (about 1 × 10^3^ host-cells and 1 × 10^3^ parasites) with a Multidrop Combi dispenser. Previously, a solution of 1.67 × 10^5^ trypomastigotes per mL in assay DMEM had been prepared and 6 μL per well dispensed in the allotted control columns that defined 100% inhibition. The 0% inhibition of parasitic growth control was obtained by leaving the assay mixture untreated in its corresponding plate columns. In all wells, the percentage of DMSO never exceeded 0.5%. Plates incubated for four days at 37°C, 5% CO_2_. The substrate used for the assay fluorescence intensity (FLINT) readout was resorufin-β-D-galactopiranoside (Sigma-Aldrich) at 5 μM per well. It was diluted in PBS supplemented with the soft detergent Igepal (Fluka) as described elsewhere^44^. Upon addition to the plates, the substrate solution was incubated for 4 h at room temperature and the signal read with the EnVision microtiter plate reader (Perkin-Elmer) using the corresponding set of filters (excitation/emission at 531/595 nm).

*Trypanosoma cruzi* intracellular imaging assay: This intracellular assay in H9c2 (rat cardiomyocyte) cells was as per Alonso-Padilla, *et al.*^11^. Briefly, H9c2 cells were seeded in T-225 flasks (225 cm^2^ culture surface; Corning Inc., NY, USA) in DMEM-10% FBS for 4 h to allow attachment. Cells were then washed once with PBS before infection. *T. cruzi* trypomastigotes, collected at days 5 to 8 after infection from LLC-MK2 parasite infected cultures, were allowed to swim out for 4 h at 37°C from a centrifuged pellet (2,500 rpm/10 min/room temperature). Trypomastigotes were then collected and counted in a CASY Cell Counter. Trypomastigotes, in supplemented DMEM, were added to H9c2 cultures in a multiplicity of infection of 1 and incubated for 18 h. Cells were washed once with PBS before incubation of the infected H9c2 monolayer with trypsin (Life-Technologies) to detach cells from the flask. Cells were counted in a CASY Cell Counter using a 150 μm capillary and their density set at 5 × 10^4^ cells per mL in supplemented assay DMEM. Infected H9c2 were dispensed into 384-well poly-lysine coated assay plates at 50 μL *per* well using a Multidrop Combi dispenser. After seeding them, the plates were incubated at 37°C, 5% CO_2_ for 72 h. Cultures were then fixed and stained by addition of 50 μL of a solution containing 8% formaldehyde and 4 μM DRAQ5 DNA dye (BioStatus, UK) per well. Plates were kept light-protected and imaged one hour later in a Perkin-Elmer Opera microscope using a 20x air objective (NA 0.4) and the following acquisition set: a 635 nm laser excitation line and a 690/50 emission detection filter for DRAQ5 detection. Five images were collected per well for reliable statistical analysis. Automated image analysis was performed with a script developed on Acapella® High Content Imaging and Analysis Software (PerkinElmer). Three outputs were provided for each sample well: (1) number of host cells nuclei to determine drug-related cytotoxicity; (2) number of amastigotes per cell as infection level measurement; and (3) percentage of infected cells per well as a second infection marker.

#### Trypanosoma brucei

Primary growth inhibition assay: This resazurin (Alamar Blue) fluorescent whole cell viability assay was based on that of Sykes & Avery^14^. Assay plates (1536 well) were prepared by dispensing 25 nL of compound solution per well using an Echo acoustic dispenser (Labcyte). For single shot assays the final compound concentration was 4.2 μM. For potency determinations, eleven-point one in three dilution curves were generated and the top concentration was 42 μM. For control samples, DMSO was pre-dispensed. *T. brucei* Lister strain 427 was cultured, harvested and counted in a CASY cell counter. A parasite suspension was prepared at a final concentration of 5 × 10^4^ cells/mL in complete HMI9 medium and 6 μL dispensed to all wells containing compounds using a Multidrop Combi dispenser. 6 μL of HMI9 media supplemented with 0.4% DMSO was dispensed to C2 wells. After 48 h of incubation at 37°C, 5% CO_2_, plates were tempered for 10 min at room temperature before addition of 2 μL of resazurin to each well at a final concentration of 50 μM in PBS. Plates were incubated for 2 h at room temperature and resorufin fluorescence was determined using a PerkinElmer ViewLux plate reader (excitation at 528 nm and emission at 590 nm).

*Ttrypanosoma brucei* growth inhibition luminescent assay: An ATP-based luminescence assay was run as confirmatory probe for the selected compounds in dose–response format, as per Sykes & Avery^16^. Pre-dispensed plates (1536 well) were prepared as described above for the fluorescence assay. *T. brucei* cultures were harvested and counted in a CASY cell counter. A parasite suspension was prepared at a final concentration of 5 × 10^4^ cells/mL in complete HMI9 medium and 6 μL was dispensed to all wells containing compounds using a Multidrop Combi dispenser. 6 μL of HMI9 media with 0.4% DMSO were dispensed to control columns. Plates were incubated for 24 h at 37°C, 5% CO_2_ then tempered for 10 min at room temperature. ATP content was quantified by adding 4 μL of CellTiter-Glo® Reagent (Promega) according to the manufacturer's instructions. Luminescence was measured using a ViewLux.

### HepG2 cytotoxicity assay

Actively growing HepG2 cells were removed from a T-175 TC flask using 5 mL Eagle's MEM (containing 10 % FBS, 1 % NEAA, 1 % penicillin/streptomycin) and dispersed in the medium by repeated pipetting. Seeding density was checked to ensure that new mono-layers were not more than 50 % confluent at the time of harvesting. Cell suspension was added to 500 mL of the same medium at a final density of 1.2 × 10^5^ cells/mL. This cell suspension was dispensed (25 μL, 3000 cells per well) into 384-well clear-bottom plates using a Multidrop Combi dispenser. Prior to addition of the cell suspension, the screening compounds (250 nL) were pre-dispensed into the plates with an Echo® liquid handler. Plates were incubated for 48 h at 37°C, 5% CO_2_. After incubation, plates equilibrated at room temperature for 30 min before proceeding to develop the luminescent signal. The signal developer, CellTiter-Glo® Reagent, was allowed to equilibrate at room temperature for 30 min and added to the plates (25 μL per well) using a Multidrop Combi dispenser. Plates were left for 10 min at room temperature for stabilization and then read using a ViewLux.

### NIH-3T3 cytotoxicity

Since *T. cruzi* is an obligatory intracellular parasite, those compounds that affect the host-cells viability would also be reported as anti-parasite hits by the previously described phenotypic assay. Therefore, a complementary assay was developed to determine the hits for NIH-3T3 cytotoxicity and/or their anti-*T. cruzi* specificity. Tissue culture surface treated assay plates (1536 well) were used. 5 μL of a 2 × 10^5^ cells per mL solution were dispensed per well (~10^3^ host cells), into plates already containing compounds which had been pre-dispensed. The 100% cells survival control was determined exposing the host-cells to a 0.5% DMSO concentration, whereas the 100% toxicity control was achieved by seeding the cells in presence of Amphotericin B at 50 μM. Plates were incubated for four days at 37°C, 5% CO_2_. The assay readout was developed by addition of 5 μL of CellTiter-Glo® Reagent following the manufacturer's instructions. Luminiscence was measured using a ViewLux.

### Data analysis

Data were normalized to percent of biological response by using positive (i.e. highest response achieved by a chemical tool compound, R_Ctrl2_) or negative (i.e. lowest response achieved in the absence of any testing compound, R_Ctrl1_) controls by using the following equation (Equation 1):
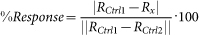
where R_x_ is the assay response measured for the compound X. R_Ctrl1_ and R_Ctrl2_ are calculated as the average of replicates in the same microtiter plate where the compound X is tested.

Assay performance statistics, such as signal to background ratio, Z′ and robust 3SD activity cutoff were calculated using templates in ActivityBase XE (IDBS, Guilford, Surrey, UK). Hit population analysis and visualization were conducted using Spotfire DecisionSite (Spotfire, Inc., Somerville, Massachusetts). pIC_50_ values were obtained using the ActivityBase XE nonlinear regression function in the full curve analysis bundle.

### Selection criteria for the anti-kinetoplastid chemical boxes

In the case of *L. donovani*, this corresponded to a pIC_50_ > 5 in the amastigote per macrophage ratio (AM/MAC) or in the percentage of infected cells (INF), a ratio of IC_50AM/MAC_/IC_50MAC_ > 5, and a ratio of IC_50AM/MAC_/IC_50HepG2_ > 10. In the case of *T. cruzi*, this comprised a pIC_50_ > 5 in the *T. cruzi* primary assay, a pIC_50_ < 5 in the HepG2 assay, a pIC_50_ < 5 in the NIH-3T3 host cell assay, a ratio of IC_50Tc_/IC_50HepG2_ > 10, and a ratio of IC_50Tc_/IC_503T3_ > 10. In the case of *T. brucei* this corresponded to a pIC_50_ > 6 in the fluorescent (primary) and luminescent (secondary) *T. brucei* whole cell assays and selectivity indexes > 10 for HepG2. In addition, all the dose–response curves were inspected and compounds were disregarded in case of aberrant fit or bizarre shape (i.e. low slope or low maximum asymptote).

### Bioinformatics analysis

In order to develop modes of action hypotheses, GSK proprietary compound screening databases were queried for historical assay data associated with active compounds from each of the three kinetoplastid species screens. The majority of these screens were against human protein targets. Thresholds for significant compound efficacy against specific human targets were defined as pIC_50_ ≥ 5 for inhibition or antagonist assays, and pEC_50_ ≥ 5 for agonist, activation or modulator assays (i.e. overall pXC_50_ ≥ 5). Human target proteins were binned by screen according to their compound hit against *L. donovani* (139 human proteins), *T. brucei* (161 human proteins) and *T. cruzi* (110 human proteins). The human target protein sequences were used as BLASTP^19^ queries to search for homologs in the respective translated reference genomes for *L. donovani* strain BPK282A1^45^, *T. brucei* strain 927/4 GUTat10.1^46^ and *T. cruzi* strain CL Brener^47^, with a cut-off E-value ≤ 1.0e-05 then subsequently confirmed by reciprocal searches against the human genome. A combined kinome phylogenetic tree was reconstructed using all annotated human kinases as well as *T. cruzi* strain 927 kinases annotated by Interpro kinase queries of TriTrypDB^4^ or reported by Urbaniak *et al.*^20^. RNAi kinase phenotypes in *T. brucei* strain 927 were annotated as death, slow or arrest according to Table 1 in Jones *et al.*^22^. Initial multiple sequence alignments were performed using the program CLUSTALW v1.7^48^ with default settings and subsequently, refined manually using the program JALVIEW^49^. Residues that could not be unambiguously aligned or that contained insertions or deletions were removed from the multiple sequence alignment prior to phylogenetic analysis. The final kinase multiple sequence alignment was 114 amino acids in length and included a combined total of 657 human and *T. brucei* kinases. We reconstructed phylogenetic trees using distance neighbor-joining (NJ) using the JTT option as implemented in the software MEGA6^50^. All trees were visualized using the program FigTree v1.4.0 (http://tree.bio.ed.ac.uk/software/figtree). Network analysis was done using KEGG reference pathways^41^ for individual parasites and general description of all analyzed genes were taken from the NCBI Gene database (http://www.ncbi.nlm.nih.gov/gene). Cytoscape was used for visualization of the compound-protein-pathway networks^42^.

### Biosafety

Experimental work with live *L. donovani* or *T. cruzi* cells were carried out following standard operating procedures in compliance with biosafety level 3 regulations (BSL3). Work with *T. brucei* was developed under biosafety level 2 procedures. HepG2 and THP-1 cells were treated according to GSK policies for management of human biological samples

## Author Contributions

J.J.M., I.P., A.R. and J.M.F. conceptualized, planned and designed the work. J.J.M. and I.P. supervised all the experimental work. I.P., J.C., I.C., J.A.-P., E.A., V.B., F.deD.-A., I.R. and A.I.B. performed the screening assays and contributed to data analysis. I.P. and M.P.M. managed the project of *Leishmania*. A.I.B. and A.K. managed the project of *T. cruzi*. I.P., M.P.M., E.A. and I.R. managed the project of *T. brucei*. D.W.G., A.R. and M.N. transferred the initial assay protocols and strains for *Leishmania, T. cruzi* and *T. brucei* screening campaigns, respectively. G.C. performed the cheminformatic analysis and contribute to data analysis, including the selection of the three chemical boxes. V.K., A.S. and J.R.B. performed the bioinformatic analysis. D.H.D. contributed to the chemical biology analysis of the target space. A.K. and M.P.M. led the final selection of compounds included in the three chemical boxes. J.J.M., I.P., J.C., E.A., A.I.B., D.H.D., J.R.B., G.C., V.K., A.S., M.P.M. and A.K. wrote different sections of the manuscript and prepared figures. I.P., J.C., J.J.M. and J.R.B. contributed critical discussions of all sections. J.C. compiled the final table containing the biological profile of compounds in the chemical boxes, and contributed to formatting of the manuscript. J.J.M. integrated individual contributions and issued the final manuscript. All authors reviewed the manuscript.

## Supplementary Material

Supplementary Information Supplemental Information

Supplementary Information Supplementary Table 2

Supplementary Information Supplementary Material Figures 3 and 4 High Resolution

## Figures and Tables

**Table 1:** Examples of proposed kinetoplastid targets for kinases based on homology to mammalian proteins used in historical assay with some examples compounds

Target class	Potential kinetoplastid target^a^			No. of chemotypes/compounds	Example structures
	*L. donovani*	*T. brucei* (RNAi phenotype)^b^	*T. cruzi*		
Ser/Thr kinases	LDBPK_050390LDBPK_071030LDBPK_081250LDBPK_100540LDBPK_130280LDBPK_150180LDBPK_151630LDBPK_160310LDBPK_170440LDBPK_170770LDBPK_180270LDBPK_181090LDBPK_191480LDBPK_200780LDBPK_211320LDBPK_211870LDBPK_220770LDBPK_242410LDBPK_261710LDBPK_272420LDBPK_280550LDBPK_292140LDBPK_292830LDBPK_300370LDBPK_300620LDBPK_303090LDBPK_320270LDBPK_331930LDBPK_332420LDBPK_340030LDBPK_343750LDBPK_344160LDBPK_360600LDBPK_360970	Tb927.9.4910Tb927.9.11030Tb927.9.14430Tb927.10.460Tb927.10.1070Tb927.10.1910Tb927.10.4990Tb927.10.5140Tb927.10.5310Tb927.10.8420Tb927.10.10350Tb927.10.12040Tb927.10.13010Tb927.10.13490Tb927.10.13780Tb927.10.14420Tb927.10.14780Tb927.10.16030Tb927.11.3140Tb927.11.4470Tb927.11.8220Tb927.3.3190Tb927.4.800Tb927.4.2500Tb927.4.3420 (S)Tb927.4.3770Tb927.5.790 (D)Tb927.5.1650Tb927.5.2820Tb927.6.2250Tb927.7.3580Tb927.7.3650Tb927.7.6220 (A)Tb927.7.6310Tb927.8.870Tb927.8.3550Tb927.8.5730 (A)Tb927.8.5950Tb927.11.14070Tb927.1.1000	Tc00.1047053398235.10Tc00.1047053506007.40Tc00.1047053504181.40Tc00.1047053504001.6Tc00.1047053508041.10Tc00.1047053503953.30Tc00.1047053508917.10Tc00.1047053510409.10Tc00.1047053511299.70Tc00.1047053510733.10Tc00.1047053511269.50Tc00.1047053511633.70Tc00.1047053506477.60Tc00.1047053510407.9Tc00.1047053509607.70Tc00.1047053509065.180Tc00.1047053509941.120Tc00.1047053508817.80Tc00.1047053508965.60Tc00.1047053503823.169Tc00.1047053510055.160Tc00.1047053509167.190Tc00.1047053510759.40Tc00.1047053509007.60Tc00.1047053509937.130Tc00.1047053510329.210Tc00.1047053511127.320Tc00.1047053508461.280Tc00.1047053511277.140Tc00.1047053508153.400Tc00.1047053506825.180Tc00.1047053507063.130Tc00.1047053506679.80Tc00.1047053508909.330Tc00.1047053507993.80Tc00.1047053511283.280	29/78	2,4-diamino-pyrimidine series (16 compounds) TCMDC-1433652-amino-4-aryl pyrimidine series (11 compounds) TCMDC-143634Amino-heterocycle-pyrimidine series (7 compounds) TCMDC-143341Azaindole series (5 compounds) TCMDC-143234Thiophene benzimidazole series (4 compounds) TCMDC-143596
Phosphatidylinositol-kinases	LDBPK_291550LDBPK_242090LDBPK_140020LDBPK_044320	Tb927.3.4020Tb927.4.1140Tb927.8.6210	Tc00.1047053510003.30Tc00.1047053510167.10	2/5	TCMDC-143352

aGene loci identifiers given in descending order for *Leishmania donovani* (LDBPK), *Trypanosoma brucei* (Tb) and *T. cruzi* (Tc).

bRNAi phenotypes scored for Arrest (A), Slow (S) and Death (D) in *Trypanosoma brucei* according to Table 1 in Jones *et al*.^22^.

**Table 2:** Some examples of proposed kinetoplastid targets for non-kinases based on homology to mammalian proteins used in historical assay with some examples compounds

Target class	Potential kinetoplastid target		No. of chemotypes/compounds	Example structures
	Protein	Gene loci^a^		
Other enzyme	Phosphosdiestearase	LDBPK_181100Tc00.1047053511269.40Tc00.1047053509805.20Tb10.389.0510	3/4	TCMDC-143547 TCMDC-143248
Proteases	Cysteine peptidases A (CBA) or C (CPC)	LDBPK_290860 (CPC)LDBPK_191460 (CBA)Tb927.6.560 (CPC)Tb927.6.960 (precursor)Tb927.6.1020 (precursor)Tc00.1047053510535.100 (CPC)	3/6	TCMDC-143238 TCMDC-143390
Cytochromes	Ferric reductase (cytochrome homology)	LDBPK_301630Tb11.02.1990Tc00.1047053507665.30	5/5	TCMDC-143560 (also a kinase inhibitor scaffold) TCMDC-143603
	Cytochrome P450 andlanosterol 14-alpha-demethylase	LDBPK_270090LDBPK_343110Tb03.27F10.920Tb11.02.4080Tc00.1047053509231.10Tc00.1047053509719.40Tc00.1047053510101.50	24/44	TCMDC-143376 TCMDC-143433
