# Supplementary material for: New Compound Sets Identified from High Throughput Phenotypic Screening Against Three Kinetoplastid Parasites: An Open Resource
Source: Sci Rep. 2015 Mar 5;5:8771. doi: 10.1038/srep08771 (PMC4350103; doi:10.1038/srep08771)
Supplement: Supplementary Information — Supplemental Information [file srep08771-s1.pdf]

New Compound Sets Identified from High Throughput Phenotypic Screening against Three Kinetoplastid Parasites: An Open Resource

Imanol Peña[1], M. Pilar Manzano[2], Juan Cantizani[2], Albane Kessler[2], Julio Alonso-Padilla[3], Ana I. Bardera[1], Emilio Alvarez[1], Gonzalo Colmenarejo[1], Ignacio Cotillo[2], Irene Roquero[1], Francisco de Dios-Anton[1], Vanessa Barroso[1], Ana Rodriguez[3], David W. Gray[4], Miguel Navarro[5], Vinod Kumar[6], Alexander Sherstnev[7], David Drewry[8], James R. Brown[6], Jose M. Fiandor[2] & J. Julio Martin[1*]

[1]Molecular Discovery Research, Tres Cantos Medicines Development Campus, GlaxoSmithKline, Tres Cantos, Spain. [2]Diseases of the Developing World (DDW), Tres Cantos Medicines Development Campus, GlaxoSmithKline, Tres Cantos, Spain. [3]Department of Microbiology, Division of Parasitology, New York University School of Medicine, New York, NY, USA. [4]Drug Discovery Unit, Division of Biological Chemistry and Drug Discovery, University of Dundee, Dundee, UK. [5]Instituto de Parasitología y Biomedicina "López-Neyra" Consejo Superior de Investigaciones Cientificas, Granada, Spain. [6]Computational Biology, Quantitative Sciences, GlaxoSmithKline, Collegeville, PA, USA. [7]Computational Biology, Quantitative Sciences, GlaxoSmithKline, Medicines Research Center, Stevenage, Hertfordshire, UK. [8]Chemical Sciences, Molecular Discovery Research, GlaxoSmithKline, Research Triangle Park, NC, USA.

[*]Corresponding author (julio.j.martin@gsk.com)

Supplementary Information

Figure 1: Outcomes from growth inhibitor high throughput screening assays for *Leishmania donovani, Trypanosoma cruzi* and *Trypanosoma brucei*. a, Distribution of growth inhibition activities for all compounds tested in the primary assay at a single compound concentration of 5 µM for *L. donovani* and *T. cruzi* assays and at 4.2 µM for the *T. brucei* assay (~1.8 million compounds distributed in 1000 activity bins). Counts representing compounds with a response above the 3SD statistical cut-off, i.e. hits, are marked in pink. **b,** Correlation plot of the confirmatory assay of select HTS hits carried out in duplicate at a 5 µM compound concentration for *L. donovani* and *T. cruzi* assays and at 4.2 µM for *T. brucei*. Each dot represents a compound. Confirmed hits are marked in green.

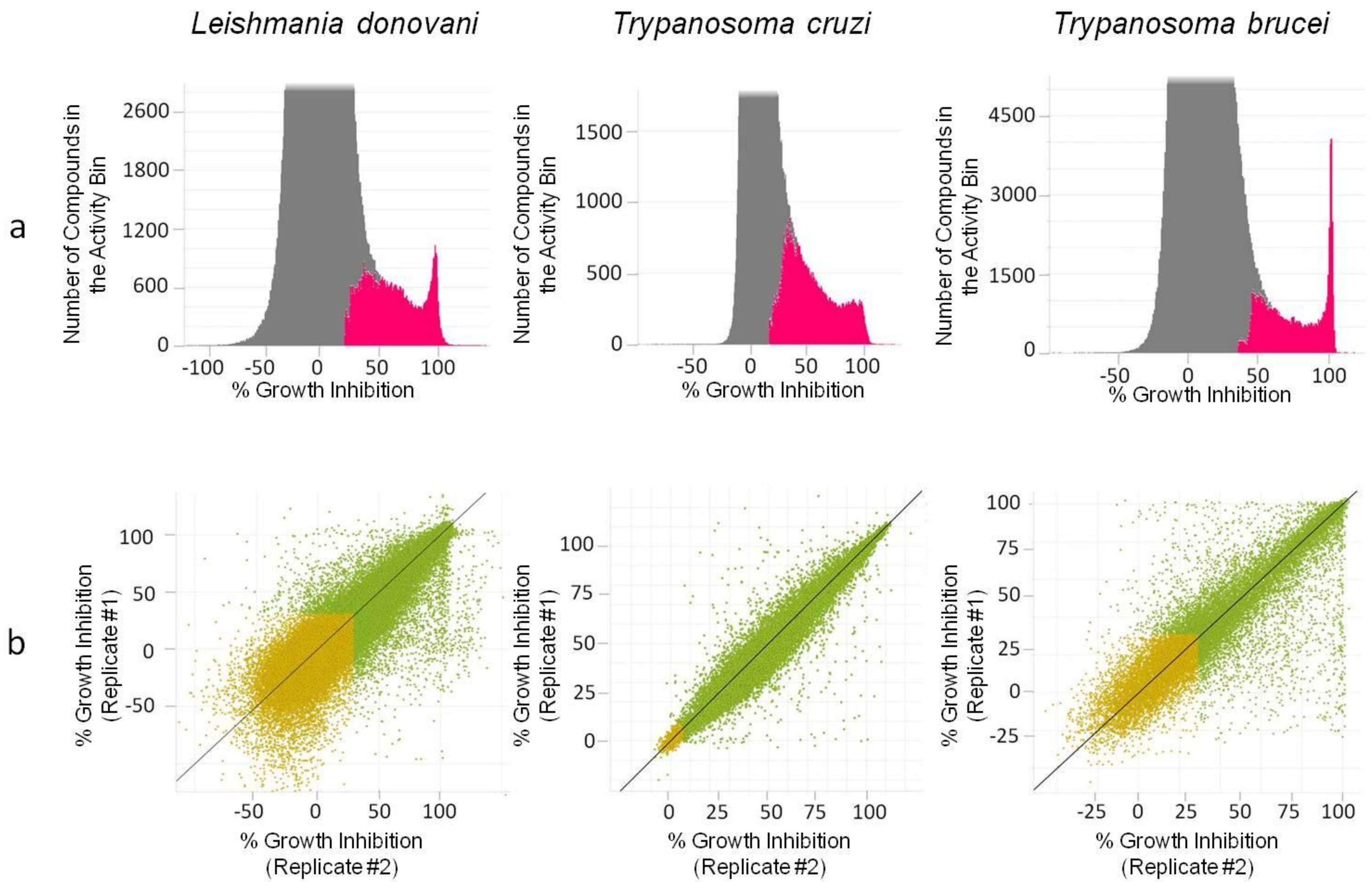

Leishmania donovani
Trypanosoma cruzi
Trypanosoma brucei
a
b
Number of Compounds in the Activity Bin
% Growth Inhibition
% Growth Inhibition (Replicate #1)
% Growth Inhibition (Replicate #2)

Figure 2: High throughput screening (HTS) progression cascade leading to the assembly of three chemical boxes of non-cytotoxic compounds active against *Leishmania donovani, Trypanosoma cruzi* and *Trypanosoma brucei*.

Abbreviations: **DR**, Dose-response; **AM**, number of amastigotes as output from imaging assay; **MAC**, number of macrophages as output from imaging assay; **AM/MAC**, ratio of amastigotes per macrophage as output from imaging assay; **INF**, percentage of infected host cell as output from imaging assay; **SI MAC**, selectivity index as ratio of IC50 versus macrophages over IC50 versus amastigotes; **SI HepG2**, selectivity index as ratio of IC50 versus HepG2 cells over IC50 versus amastigotes; **SI NIH 3T3**, selectivity index as ratio of IC50 versus NIH 3T3 cells over IC50 versus amastigotes; **QC**, quality control; **cPFI**, calculated Property Forecast Index; ***T. cruzi*-1ry**, *Trypanosoma cruzi* viability assay using β-GAL activity; **NIH 3T3-2ry**, NIH 3T3 cell line viability assay using luminescence; ***T. brucei*-1ry**, pIC50 *Trypanosoma brucei* viability assay using fluorescence intensity; ***T. brucei*-2ry**, *Trypanosoma brucei* viability assay using luminescence.

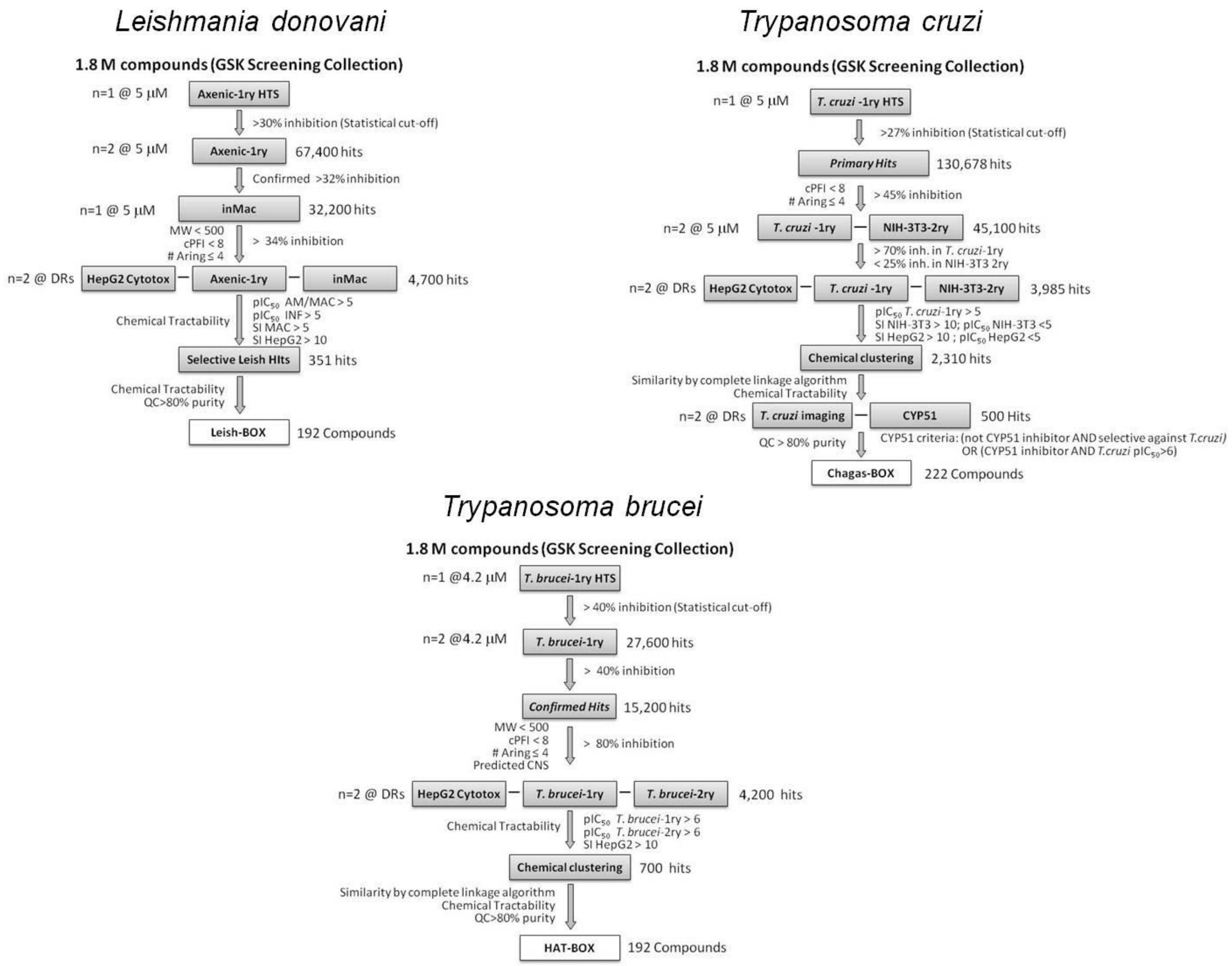
Leishmania donovani
1.8 M compounds (GSK Screening Collection)
n=1 @ 5 μM
Axenic-1ry HTS
>30% inhibition (Statistical cut-off)
n=2 @ 5 μM
Axenic-1ry
67,400 hits
Confirmed >32% inhibition
n=1 @ 5 μM
inMac
32,200 hits
MW < 500
cPFI < 8
Aring ≤ 4
> 34% inhibition
n=2 @ DRs
HepG2 Cytotox
Axenic-1ry
inMac
4,700 hits
Chemical Tractability
pIC50 AM/MAC > 5
pIC50 INF > 5
SI MAC > 5
SI HepG2 > 10
Selective Leish HIts
351 hits
Chemical Tractability
QC>80% purity
Leish-BOX
192 Compounds
Trypanosoma cruzi
1.8 M compounds (GSK Screening Collection)
n=1 @ 5 μM
T. cruzi -1ry HTS
>27% inhibition (Statistical cut-off)
Primary Hits
130,678 hits
cPFI < 8
Aring ≤ 4
> 45% inhibition
n=2 @ 5 μM
T. cruzi -1ry
NIH-3T3-2ry
45,100 hits
> 70% inh. in T. cruzi-1ry
< 25% inh. in NIH-3T3 2ry
n=2 @ DRs
HepG2 Cytotox
T. cruzi -1ry
NIH-3T3-2ry
3,985 hits
pIC50 T. cruzi-1ry > 5
SI NIH-3T3 > 10; pIC50 NIH-3T3 <5
SI HepG2 > 10 ; pIC50 HepG2 <5
Chemical clustering
2,310 hits
Similarity by complete linkage algorithm
Chemical Tractability
n=2 @ DRs
T. cruzi imaging
CYP51
500 Hits
QC > 80% purity
CYP51 criteria: (not CYP51 inhibitor AND selective against T.cruzi)
OR (CYP51 inhibitor AND T.cruzi pIC50>6)
Chagas-BOX
222 Compounds
Trypanosoma brucei
1.8 M compounds (GSK Screening Collection)
n=1 @4.2 μM
T. brucei-1ry HTS
> 40% inhibition (Statistical cut-off)
n=2 @4.2 μM
T. brucei-1ry
27,600 hits
> 40% inhibition
Confirmed Hits
15,200 hits
MW < 500
cPFI < 8
Aring ≤ 4
Predicted CNS
> 80% inhibition
n=2 @ DRs
HepG2 Cytotox
T. brucei-1ry
T. brucei-2ry
4,200 hits
Chemical Tractability
pIC50 T. brucei-1ry > 6
pIC50 T. brucei-2ry > 6
SI HepG2 > 10
Chemical clustering
700 hits
Similarity by complete linkage algorithm
Chemical Tractability
QC>80% purity
HAT-BOX
192 Compounds

Figure 3: Kinetoplastidal activity did not correlate well between orthogonal assays against *L. donovani*, but good correlations were seen for assays against *T. cruzi* and *T. brucei*. a, *L. donovani* pIC_{50} values for amastigote per macrophage readout from intracellular imaging assay *versus* fluorescence intensity from axenic assay. **b,** *T. cruzi* pIC_{50} values for amastigote per cell from the intracellular imaging assay *versus.* fluorescence intensity from the intracellular beta-galacosidase reporter assay. **c,** *T. brucei* pIC_{50} values from fluorescent resazurin-based assay *versus* luminescent ATP-based assay. Straight lines correspond to y=x equation.

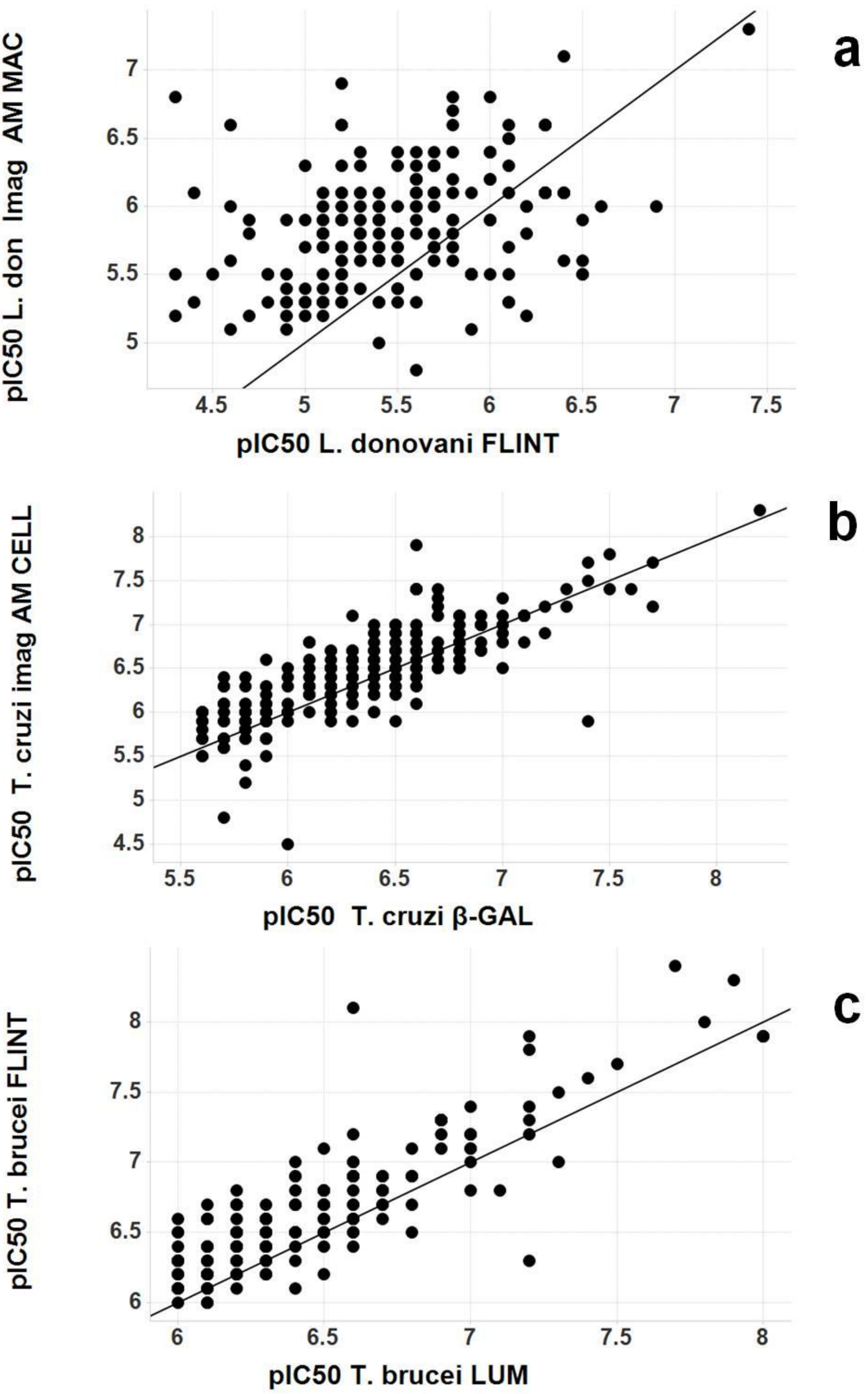
a
pIC50 L. don Imag AM MAC
pIC50 L. donovani FLINT
b
pIC50 T. cruzi imag AM CELL
pIC50 T. cruzi β-GAL
c
pIC50 T. brucei FLINT
pIC50 T. brucei LUM

Figure 4: Flagging putative CYP51-acting compounds with anti-parasitidal activity against *T. cruzi*. Scatter plot for all compounds in the *T. cruzi* chemical box. x-axis represents pIC_{50} values in the *T. cruzi* CYP51 biochemical assay; y-axis represents pIC_{50} values in the amastigotes per cell readout from the imaging assay.

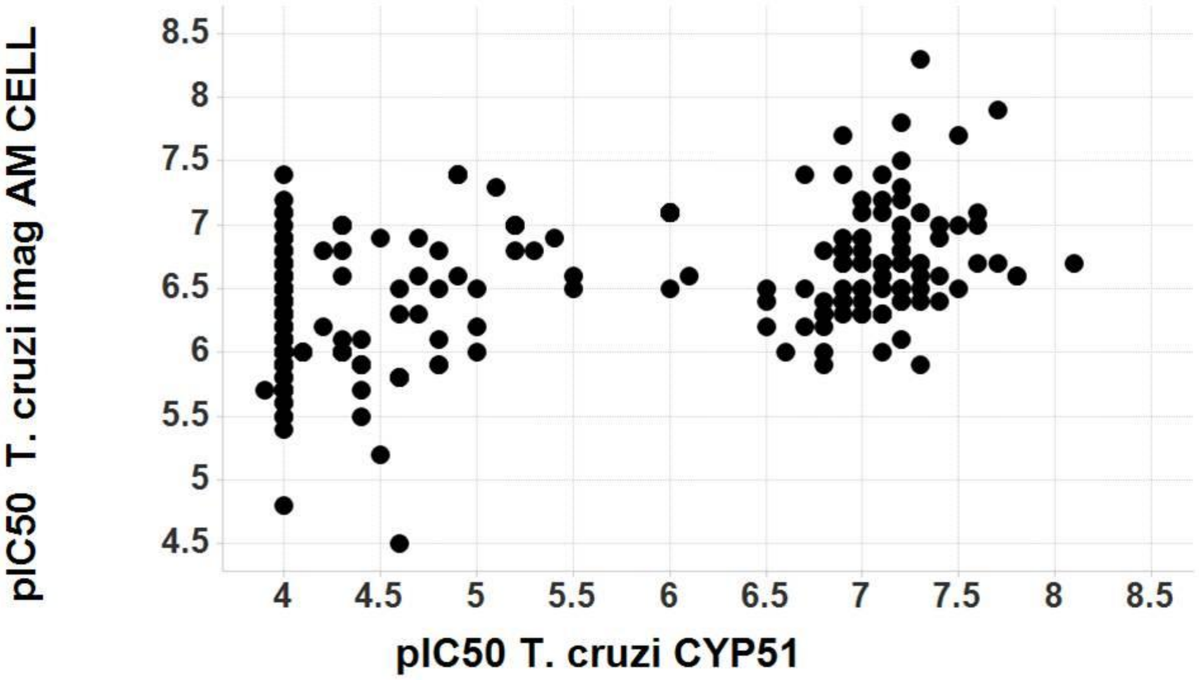

Figure 5: Compounds composing the *L. donovani* and *T. cruzi* chemical boxes are able to completely eliminate parasites in the infected host cells. Scatter plot of pIC_{50} values for the percent of infected host cell readout *versus* pIC_{50} values for the total number of amastigotes readout from the imaging assays in **a,** *L. donovani* and **b,** *T. cruzi*. Each symbol represents a compound. Straight lines correspond to y=x equation.

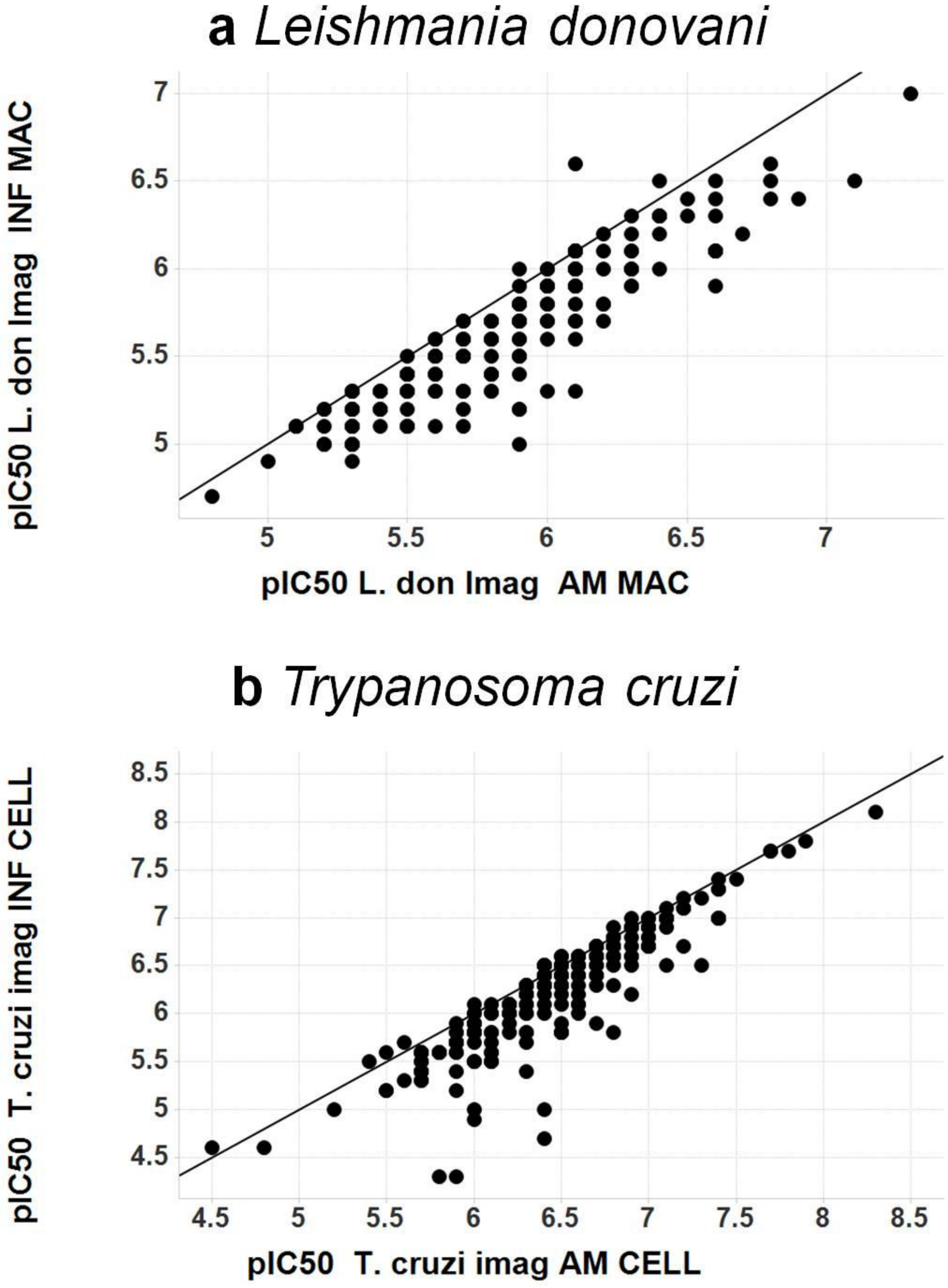

a Leishmania donovani
pIC50 L. don Imag INF MAC
pIC50 L. don Imag AM MAC
7
6.5
6
5.5
5
b Trypanosoma cruzi
pIC50 T. cruzi imag INF CELL
pIC50 T. cruzi imag AM CELL
4.5
8
8.5

Figure 6: Distribution of physicochemical descriptors of the *Leishmania* (ld), HAT (tb) and Chagas (tc) chemical boxes in comparison with the whole GSK compound collection (GSK). Box plots for **a,** clogP, **b,** molecular weight (Da) and **c,** number of aromatic rings.

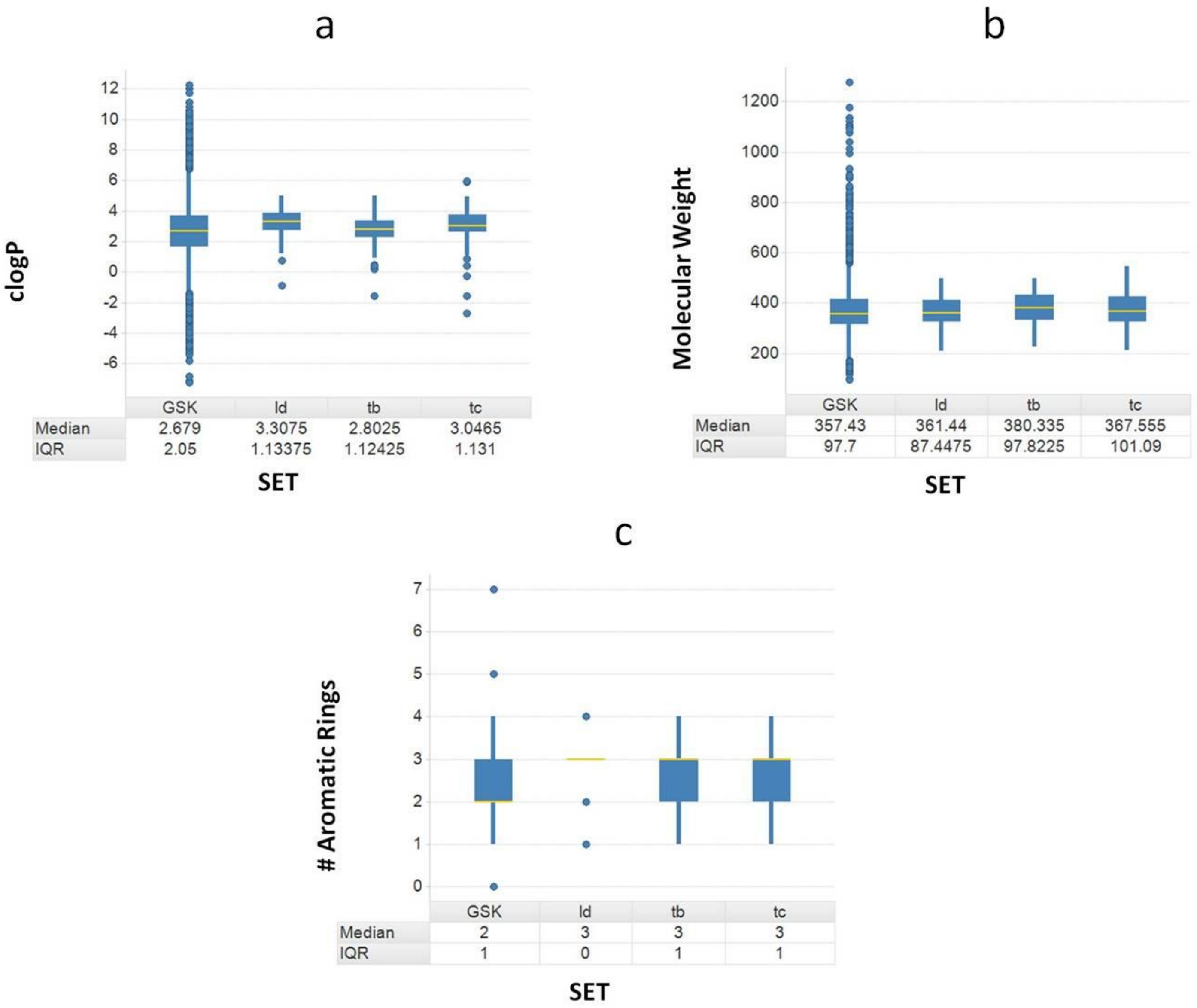

	GSK	ld	tb	tc
Median	2.679	3.3075	2.8025	3.0465
IQR	2.05	1.13375	1.12425	1.131

	GSK	ld	tb	tc
Median	357.43	361.44	380.335	367.555
IQR	97.7	87.4475	97.8225	101.09

	GSK	ld	tb	tc
Median	2	3	3	3
IQR	1	0	1	1

Table 1: Small molecule screening campaign data of the *Leishmania donovani*, *Trypanosoma cruzi* and *Trypanosoma brucei* growth inhibitor HTS

Category	Parameter	Leish HTS	Chagas HTS	HAT HTS
Assay	Type of assay	*in vitro*; phenotypic; parasite grow	*in vitro*; phenotypic; parasite grow	*in vitro*; phenotypic; parasite grow
	Target	*Leishmania donovani*	intracellular *Trypanosoma cruzi*	*Trypanosoma brucei brucei*
	Primary measurement	Fluorescence intensity of produced Resorufin from reduction of non-fluorescent Resazurin by viable cells	Fluorescence intensity of produced Resorufin from reduction of non-fluorescent Resazurin by viable cells	Fluorescence intensity of produced Resorufin from reduction of non-fluorescent Resazurin by viable cells
	Key reagents	*L. d. donovani* (MHOM/ET/67/HU3 strain), Resazurin	*T. cruzi* -β-gal (Tulahuen strain, clone C4), NIH-3T3 MEF cells,	*T. b. brucei* (Lister 427 strain) Resazurin
	Assay protocol	See Methods Section	See Methods Section	See Methods Section
Library	Library size	Approximately 1.8 million	Approximately 1.8 million	Approximately 1.8 million
	Library composition	Diversity collection	Diversity collection	Diversity collection
	Source	GSK	GSK	GSK
Screen	Format	1536-well plates (Greiner catalog #783096)	1536-well plates (Greiner catalog #782092)	1536-well plates (Greiner catalog #782092)
	Concentration(s) tested	5 µM	5 µM	4.2 µM
	Plate controls	DMSO	DMSO	DMSO
	Reagent/ compound dispensing system	Compound Dispensation: (GSK Sample Management Technology group) Test CMPD, DMSO control: 30 nL/well using Echo® Acoustic Dispenser (Labcyte Inc., Sunnyvale, CA). All Subsequent Assay Reagent Dispensations: Multidrop Combi (Thermo Scientific, Waltham MA)	Compound Dispensation: (GSK Sample Management Technology group) Test CMPD, DMSO control: 30 nL/well using Echo® Acoustic Dispenser (Labcyte Inc., Sunnyvale, CA). All Subsequent Assay Reagent Dispensations: Multidrop Combi (Thermo Scientific, Waltham MA)	Compound Dispensation: (GSK Sample Management Technology group) Test CMPD, DMSO control: 30 nL/well using Echo® Acoustic Dispenser (Labcyte Inc., Sunnyvale, CA). All Subsequent Assay Reagent Dispensations: Multidrop Combi (Thermo Scientific, Waltham MA)
	Detection instrument and software	Plate Readers: Envision (Perkin Elmer, Inc., Waltham MA)	Plate Readers: Envision (Perkin Elmer, Inc., Waltham MA)	Plate Readers: Envision (Perkin Elmer, Inc., Waltham MA)
	Assay validation/QC	Average Z' value = 0.71 (n=1393 plates in entire HTS primary screen).	Average Z' value = 0.85 (n=1298 plates in entire HTS primary screen).	Average Z' value = 0.72 (n=1194 plates in entire HTS primary screen).
	Correction factors	Systematic Error correction and Pattern Recognition Tool	Systematic Error correction and Pattern Recognition Tool	Systematic Error correction and Pattern Recognition Tool
	Normalization	%Response= (RCtrl1-Rx)/(RCtrl1-RCtrl2) · 100	%Response= (RCtrl1-Rx)/(RCtrl1-RCtrl2) · 100	%Response= (RCtrl1-Rx)/(RCtrl1-RCtrl2) · 100
	Additional comments	Rx is the assay response measured for the compound X. RCtrl1 and RCtrl2 are calculated as the average of replicates in the same microtiter plate where the compound X is tested.	Rx is the assay response measured for the compound X. RCtrl1 and RCtrl2 are calculated as the average of replicates in the same microtiter plate where the compound X is tested.	Rx is the assay response measured for the compound X. RCtrl1 and RCtrl2 are calculated as the average of replicates in the same microtiter plate where the compound X is tested.
Post-HTS analysis	Hit criteria	Statistical Cut-off: 32%Inhibition average in primary	Statistical Cut-off: 27%Inhibition average in primary	Statistical Cut-off: 40%Inhibition average in primary
	Hit rate	3.7% (50% confirmation rate: 32K hits confirmed)	7.70% (reduced to 2.5% by biological and physchem properties)	1.5% (56% Confirmation Rate: 15K hits confirmed)
	Additional assay(s)	Intra-macrophage Ld Imaging, HepG2	Intra-cardiomyocyte Tc Imaging, HepG2, 3T3	Tbb Lumi, HepG2
	Confirmation of hit purity and structure	>80% Purity	>80% Purity	>80% Purity

Table 2: Biolological and Physicochemical Profiling of the compounds constituting the three kinetoplastid boxes.
