# Supplementary material for: New Compound Sets Identified from High Throughput Phenotypic Screening Against Three Kinetoplastid Parasites: An Open Resource
Source: Sci Rep. 2015 Mar 5;5:8771. doi: 10.1038/srep08771 (PMC4350103; doi:10.1038/srep08771)
Supplement: Supplementary Information — Supplementary Material Figures 3 and 4 High Resolution [file srep08771-s3.pdf]

New Compound Sets Identified from High Throughput Phenotypic Screening against Three Kinetoplastid Parasites: An Open Resource

Imanol Peña[1], M. Pilar Manzano[2], Juan Cantizani[2], Albane Kessler[2], Julio Alonso-Padilla[3], Ana I. Bardera[1], Emilio Alvarez[1], Gonzalo Colmenarejo[1], Ignacio Cotillo[2], Irene Roquero[1], Francisco de Dios-Anton[1], Vanessa Barroso[1], Ana Rodriguez[3], David W. Gray[4], Miguel Navarro[5], Vinod Kumar[6], Alexander Sherstnev[7], David Drewry[8], James R. Brown[6], Jose M. Fiandor[2] & J. Julio Martin[1*]

[1]Molecular Discovery Research, Tres Cantos Medicines Development Campus, GlaxoSmithKline, Tres Cantos, Spain. [2]Diseases of the Developing World (DDW), Tres Cantos Medicines Development Campus, GlaxoSmithKline, Tres Cantos, Spain. [3]Department of Microbiology, Division of Parasitology, New York University School of Medicine, New York, NY, USA. [4]Drug Discovery Unit, Division of Biological Chemistry and Drug Discovery, University of Dundee, Dundee, UK. [5]Instituto de Parasitología y Biomedicina "López-Neyra" Consejo Superior de Investigaciones Cientificas, Granada, Spain. [6]Computational Biology, Quantitative Sciences, GlaxoSmithKline, Collegeville, PA, USA. [7]Computational Biology, Quantitative Sciences, GlaxoSmithKline, Medicines Research Center, Stevenage, Hertfordshire, UK. [8]Chemical Sciences, Molecular Discovery Research, GlaxoSmithKline, Research Triangle Park, NC, USA.

[*]Corresponding author (julio.j.martin@gsk.com)

Supplementary Information

Figure 3. Neighbor-joining phylogenetic tree of combined human and *Trypanosoma brucei* str. 927 kinomes.

Human kinase targets with putative compounds are colored and prefixed (T_red); all human kinases are colored black. *T. brucei* kinases are colored and prefixed for the RNAi phenotypes death (D_blue), arrest (A_dark green) or slow (S_orange) according to Table 1 in Jones *et al.*[22]. All other *T. brucei* kinases are colored violet. A few key clusters of human target and essential *T. brucei* kinases are labeled. The size and color of circles on the nodes represent support in 1000 bootstrap replicates.

22. Jones, N.G. et al. Regulators of *Trypanosoma brucei* cell cycle progression and differentiation identified using a kinome-wide RNAi screen. *PLoS Pathog.* **10**, e1003886 (2014)

Note: the figure below is a high resolution version of Figure 3 in the main manuscript. It can be zoomed in at your convenience

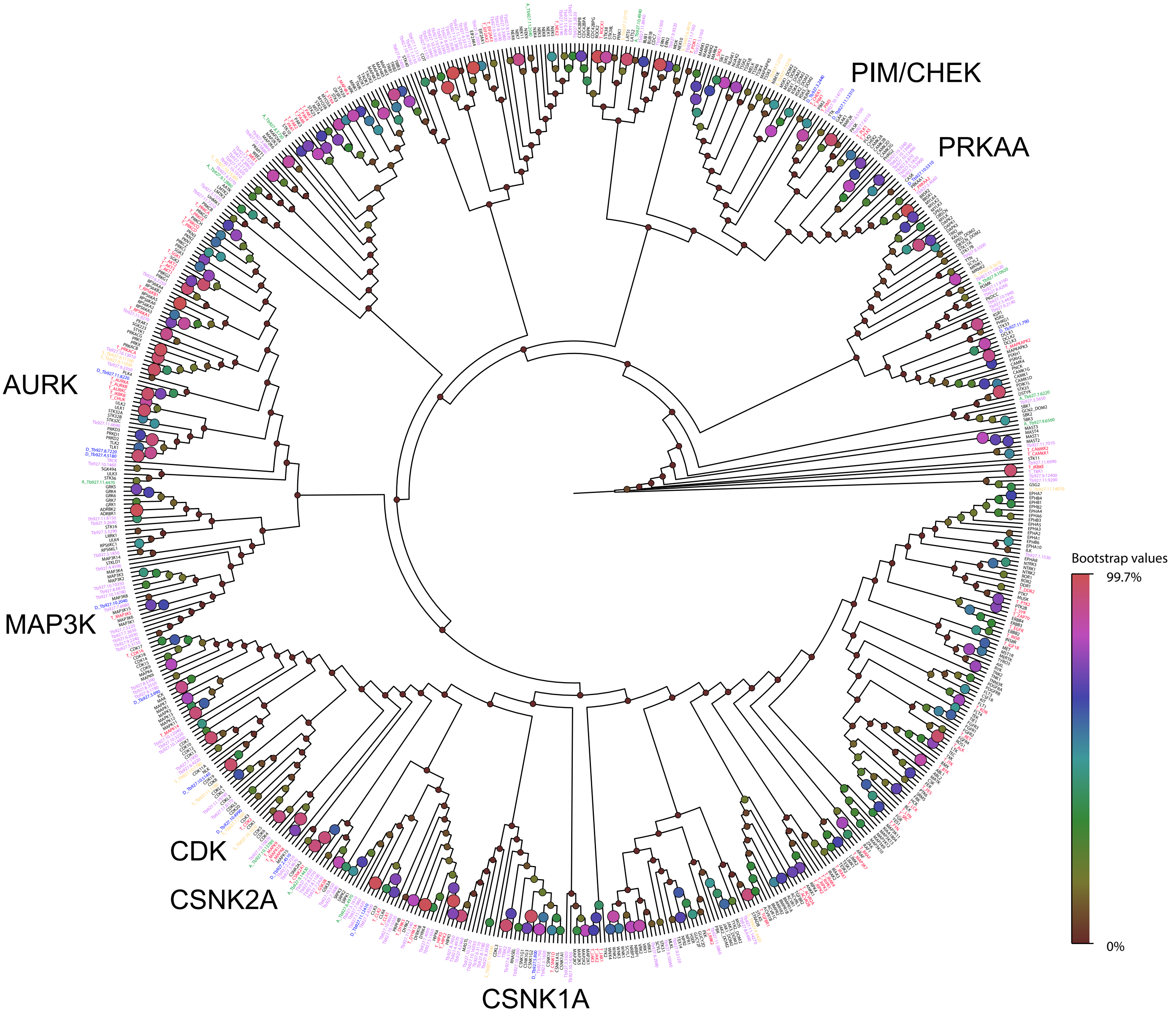
PIM/CHEK
PRKAA
AURK
MAP3K
CDK
CSNK2A
CSNK1A
Bootstrap values
99.7%
0%

Supplementary Information

Figure 4. Pathway network analysis of combined and individual kinetoplastid target hypotheses.

Pathways were assigned using KEGG and visualized using Cytoscape[42]. Proteins of three species are grouped and colored according to general functional groups (kinases, proteases, other enzymes and cytochromes).

42. Shannon, P. et al. Cytoscape: a software environment for integrated models of biomolecular interaction networks. *Genome Res.* **13**, 2498–504 (2013).

Note: the figure below is a high resolution version of Figure 4 in the main manuscript. It can be zoomed in at your convenience

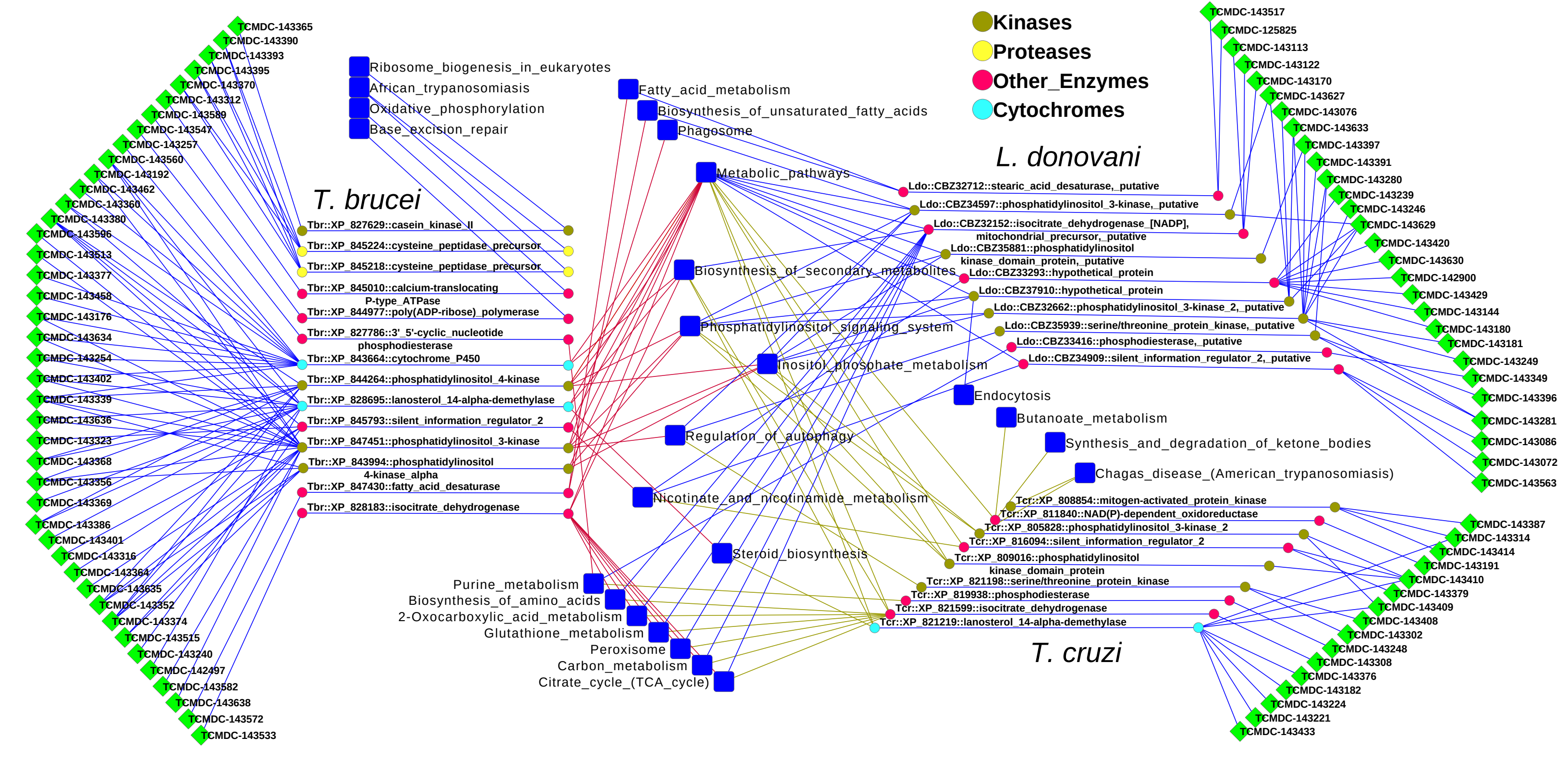
Kinases
Proteases
Other_Enzymes
Cytochromes
T. brucei
L. donovani
T. cruzi
TCMDC-143365
TCMDC-143390
TCMDC-143393
TCMDC-143395
TCMDC-143370
TCMDC-143312
TCMDC-143589
TCMDC-143547
TCMDC-143257
TCMDC-143560
TCMDC-143192
TCMDC-143462
TCMDC-143360
TCMDC-143380
TCMDC-143596
TCMDC-143513
TCMDC-143377
TCMDC-143458
TCMDC-143176
TCMDC-143634
TCMDC-143254
TCMDC-143402
TCMDC-143339
TCMDC-143636
TCMDC-143323
TCMDC-143368
TCMDC-143356
TCMDC-143369
TCMDC-143386
TCMDC-143401
TCMDC-143316
TCMDC-143364
TCMDC-143635
TCMDC-143352
TCMDC-143374
TCMDC-143515
TCMDC-143240
TCMDC-142497
TCMDC-143582
TCMDC-143638
TCMDC-143572
TCMDC-143533
Ribosome_biogenesis_in_eukaryotes
African_trypanosomiasis
Oxidative_phosphorylation
Base_excision_repair
Fatty_acid_metabolism
Biosynthesis_of_unsaturated_fatty_acids
Phagosome
Metabolic_pathways
Biosynthesis_of_secondary_metabolites
Phosphatidylinositol_signaling_system
Inositol_phosphate_metabolism
Endocytosis
Butanoate_metabolism
Synthesis_and_degradation_of_ketone_bodies
Chagas_disease_(American_trypanosomiasis)
Regulation_of_autophagy
Nicotinate_and_nicotinamide_metabolism
Steroid_biosynthesis
Purine_metabolism
Biosynthesis_of_amino_acids
2-Oxocarboxylic_acid_metabolism
Glutathione_metabolism
Peroxisome
Carbon_metabolism
Citrate_cycle_(TCA_cycle)
Tbr::XP_827629::casein_kinase_II
Tbr::XP_845224::cysteine_peptidase_precursor
Tbr::XP_845218::cysteine_peptidase_precursor
Tbr::XP_845010::calcium-translocating P-type_ATPase
Tbr::XP_844977::poly(ADP-ribose)_polymerase
Tbr::XP_827786::3'_5'-cyclic_nucleotide phosphodiesterase
Tbr::XP_843664::cytochrome_P450
Tbr::XP_844264::phosphatidylinositol_4-kinase
Tbr::XP_828695::lanosterol_14-alpha-demethylase
Tbr::XP_845793::silent_information_regulator_2
Tbr::XP_847451::phosphatidylinositol_3-kinase
Tbr::XP_843994::phosphatidylinositol 4-kinase_alpha
Tbr::XP_847430::fatty_acid_desaturase
Tbr::XP_828183::isocitrate_dehydrogenase
Ldo::CBZ32712::stearic_acid_desaturase,_putative
Ldo::CBZ34597::phosphatidylinositol_3-kinase,_putative
Ldo::CBZ32152::isocitrate_dehydrogenase_[NADP], mitochondrial_precursor,_putative
Ldo::CBZ35881::phosphatidylinositol kinase_domain_protein,_putative
Ldo::CBZ33293::hypothetical_protein
Ldo::CBZ37910::hypothetical_protein
Ldo::CBZ32662::phosphatidylinositol_3-kinase_2,_putative
Ldo::CBZ35939::serine/threonine_protein_kinase,_putative
Ldo::CBZ33416::phosphodiesterase,_putative
Ldo::CBZ34909::silent_information_regulator_2,_putative
TCMDC-143517
TCMDC-125825
TCMDC-143113
TCMDC-143122
TCMDC-143170
TCMDC-143627
TCMDC-143076
TCMDC-143633
TCMDC-143397
TCMDC-143391
TCMDC-143280
TCMDC-143239
TCMDC-143246
TCMDC-143629
TCMDC-143420
TCMDC-143630
TCMDC-142900
TCMDC-143429
TCMDC-143144
TCMDC-143180
TCMDC-143181
TCMDC-143249
TCMDC-143349
TCMDC-143396
TCMDC-143281
TCMDC-143086
TCMDC-143072
TCMDC-143563
Tcr::XP_808854::mitogen-activated_protein_kinase
Tcr::XP_811840::NAD(P)-dependent_oxidoreductase
Tcr::XP_805828::phosphatidylinositol_3-kinase_2
Tcr::XP_816094::silent_information_regulator_2
Tcr::XP_809016::phosphatidylinositol kinase_domain_protein
Tcr::XP_821198::serine/threonine_protein_kinase
Tcr::XP_819938::phosphodiesterase
Tcr::XP_821599::isocitrate_dehydrogenase
Tcr::XP_821219::lanosterol_14-alpha-demethylase
TCMDC-143387
TCMDC-143314
TCMDC-143414
TCMDC-143191
TCMDC-143410
TCMDC-143379
TCMDC-143409
TCMDC-143408
TCMDC-143302
TCMDC-143248
TCMDC-143308
TCMDC-143376
TCMDC-143182
TCMDC-143224
TCMDC-143221
TCMDC-143433

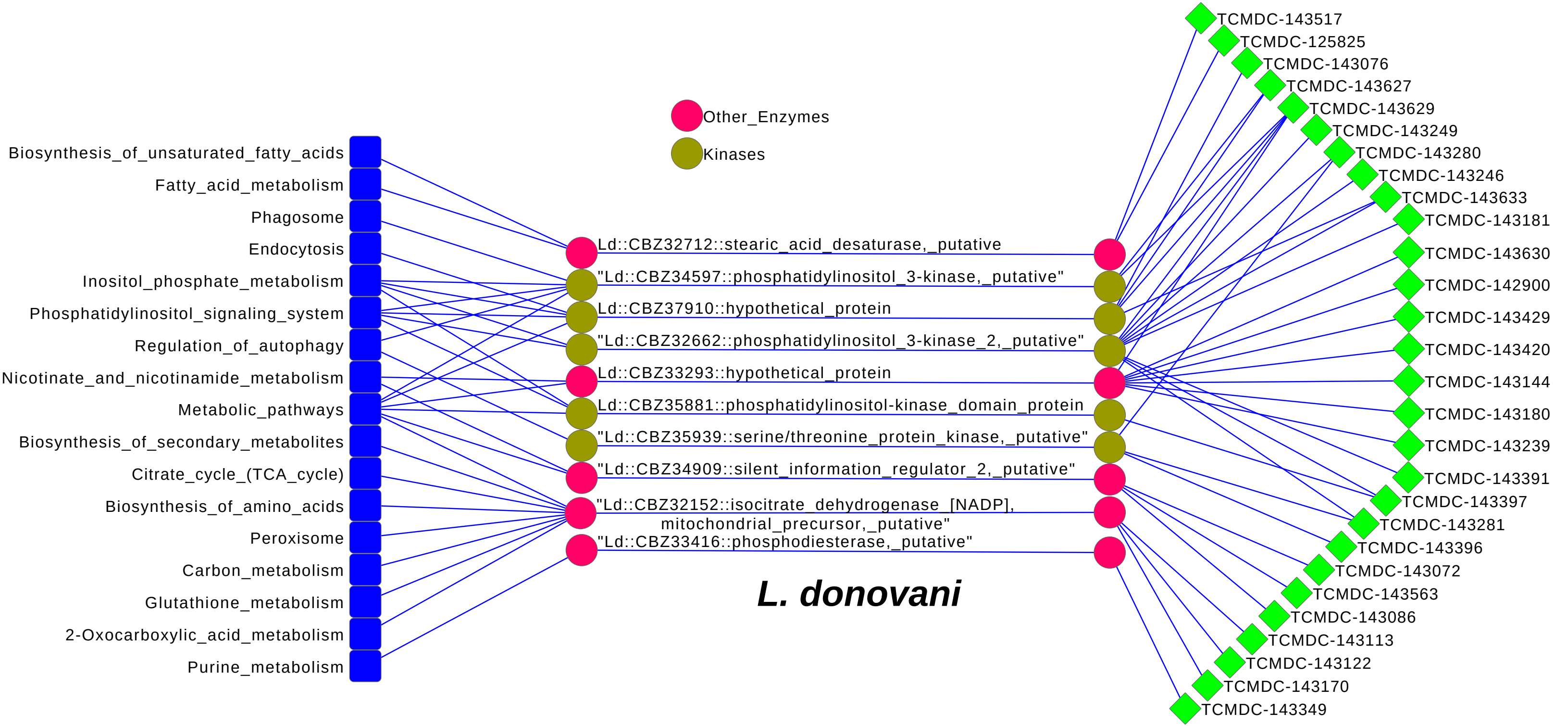

Other_Enzymes
Kinases
Biosynthesis_of_unsaturated_fatty_acids
Fatty_acid_metabolism
Phagosome
Endocytosis
Inositol_phosphate_metabolism
Phosphatidylinositol_signaling_system
Regulation_of_autophagy
Nicotinate_and_nicotinamide_metabolism
Metabolic_pathways
Biosynthesis_of_secondary_metabolites
Citrate_cycle_(TCA_cycle)
Biosynthesis_of_amino_acids
Peroxisome
Carbon_metabolism
Glutathione_metabolism
2-Oxocarboxylic_acid_metabolism
Purine_metabolism
Ld::CBZ32712::stearic_acid_desaturase,_putative
"Ld::CBZ34597::phosphatidylinositol_3-kinase,_putative"
Ld::CBZ37910::hypothetical_protein
"Ld::CBZ32662::phosphatidylinositol_3-kinase_2,_putative"
Ld::CBZ33293::hypothetical_protein
Ld::CBZ35881::phosphatidylinositol-kinase_domain_protein
"Ld::CBZ35939::serine/threonine_protein_kinase,_putative"
"Ld::CBZ34909::silent_information_regulator_2,_putative"
"Ld::CBZ32152::isocitrate_dehydrogenase_[NADP],
mitochondrial_precursor,_putative"
"Ld::CBZ33416::phosphodiesterase,_putative"
L. donovani
TCMDC-143517
TCMDC-125825
TCMDC-143076
TCMDC-143627
TCMDC-143629
TCMDC-143249
TCMDC-143280
TCMDC-143246
TCMDC-143633
TCMDC-143181
TCMDC-143630
TCMDC-142900
TCMDC-143429
TCMDC-143420
TCMDC-143144
TCMDC-143180
TCMDC-143239
TCMDC-143391
TCMDC-143397
TCMDC-143281
TCMDC-143396
TCMDC-143072
TCMDC-143563
TCMDC-143086
TCMDC-143113
TCMDC-143122
TCMDC-143170
TCMDC-143349

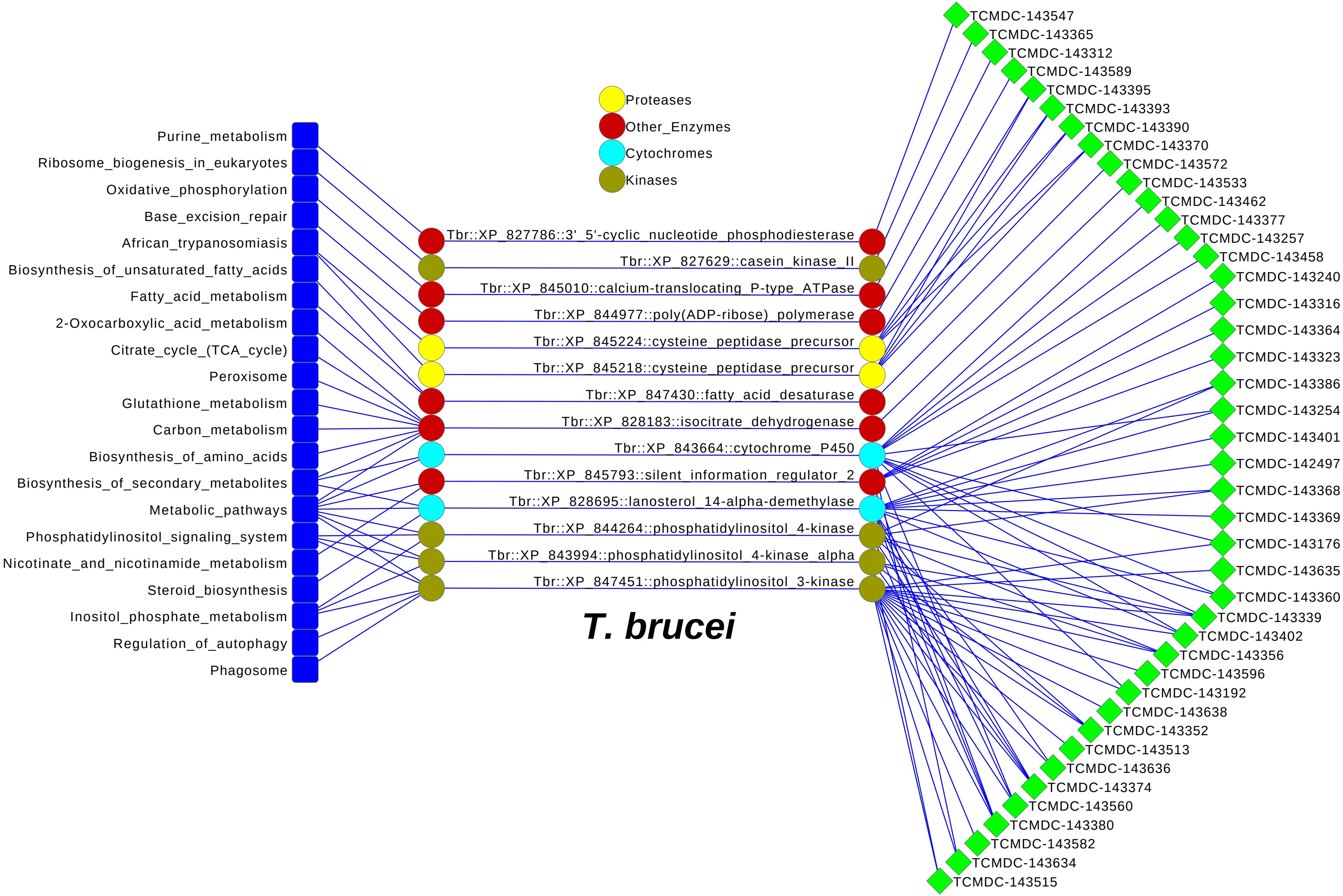

Proteases
Other_Enzymes
Cytochromes
Kinases
Purine_metabolism
Ribosome_biogenesis_in_eukaryotes
Oxidative_phosphorylation
Base_excision_repair
African_trypanosomiasis
Biosynthesis_of_unsaturated_fatty_acids
Fatty_acid_metabolism
2-Oxocarboxylic_acid_metabolism
Citrate_cycle_(TCA_cycle)
Peroxisome
Glutathione_metabolism
Carbon_metabolism
Biosynthesis_of_amino_acids
Biosynthesis_of_secondary_metabolites
Metabolic_pathways
Phosphatidylinositol_signaling_system
Nicotinate_and_nicotinamide_metabolism
Steroid_biosynthesis
Inositol_phosphate_metabolism
Regulation_of_autophagy
Phagosome
Tbr::XP_827786::3'_5'-cyclic_nucleotide_phosphodiesterase
Tbr::XP_827629::casein_kinase_II
Tbr::XP_845010::calcium-translocating_P-type_ATPase
Tbr::XP_844977::poly(ADP-ribose)_polymerase
Tbr::XP_845224::cysteine_peptidase_precursor
Tbr::XP_845218::cysteine_peptidase_precursor
Tbr::XP_847430::fatty_acid_desaturase
Tbr::XP_828183::isocitrate_dehydrogenase
Tbr::XP_843664::cytochrome_P450
Tbr::XP_845793::silent_information_regulator_2
Tbr::XP_828695::lanosterol_14-alpha-demethylase
Tbr::XP_844264::phosphatidylinositol_4-kinase
Tbr::XP_843994::phosphatidylinositol_4-kinase_alpha
Tbr::XP_847451::phosphatidylinositol_3-kinase
T. brucei
TCMDC-143547
TCMDC-143365
TCMDC-143312
TCMDC-143589
TCMDC-143395
TCMDC-143393
TCMDC-143390
TCMDC-143370
TCMDC-143572
TCMDC-143533
TCMDC-143462
TCMDC-143377
TCMDC-143257
TCMDC-143458
TCMDC-143240
TCMDC-143316
TCMDC-143364
TCMDC-143323
TCMDC-143386
TCMDC-143254
TCMDC-143401
TCMDC-142497
TCMDC-143368
TCMDC-143369
TCMDC-143176
TCMDC-143635
TCMDC-143360
TCMDC-143339
TCMDC-143402
TCMDC-143356
TCMDC-143596
TCMDC-143192
TCMDC-143638
TCMDC-143352
TCMDC-143513
TCMDC-143636
TCMDC-143374
TCMDC-143560
TCMDC-143380
TCMDC-143582
TCMDC-143634
TCMDC-143515

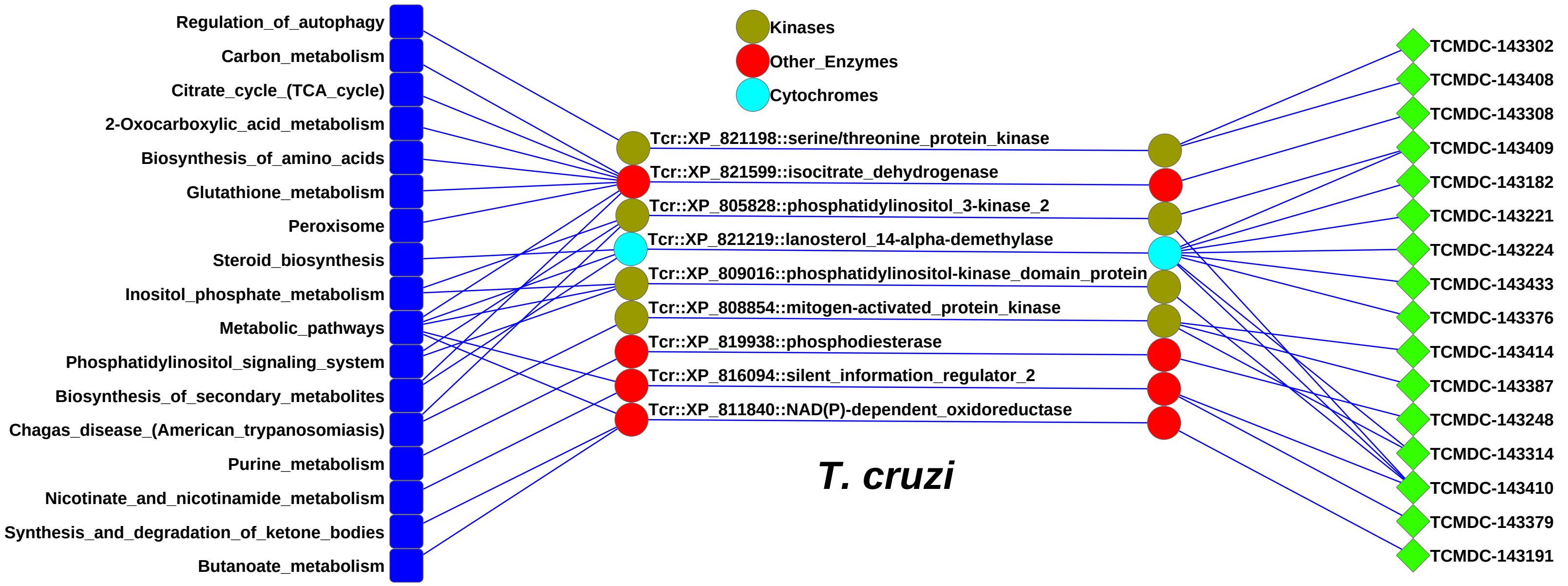
Kinases
Other_Enzymes
Cytochromes
Regulation_of_autophagy
Carbon_metabolism
Citrate_cycle_(TCA_cycle)
2-Oxocarboxylic_acid_metabolism
Biosynthesis_of_amino_acids
Glutathione_metabolism
Peroxisome
Steroid_biosynthesis
Inositol_phosphate_metabolism
Metabolic_pathways
Phosphatidylinositol_signaling_system
Biosynthesis_of_secondary_metabolites
Chagas_disease_(American_trypanosomiasis)
Purine_metabolism
Nicotinate_and_nicotinamide_metabolism
Synthesis_and_degradation_of_ketone_bodies
Butanoate_metabolism
Tcr::XP_821198::serine/threonine_protein_kinase
Tcr::XP_821599::isocitrate_dehydrogenase
Tcr::XP_805828::phosphatidylinositol_3-kinase_2
Tcr::XP_821219::lanosterol_14-alpha-demethylase
Tcr::XP_809016::phosphatidylinositol-kinase_domain_protein
Tcr::XP_808854::mitogen-activated_protein_kinase
Tcr::XP_819938::phosphodiesterase
Tcr::XP_816094::silent_information_regulator_2
Tcr::XP_811840::NAD(P)-dependent_oxidoreductase
T. cruzi
TCMDC-143302
TCMDC-143408
TCMDC-143308
TCMDC-143409
TCMDC-143182
TCMDC-143221
TCMDC-143224
TCMDC-143433
TCMDC-143376
TCMDC-143414
TCMDC-143387
TCMDC-143248
TCMDC-143314
TCMDC-143410
TCMDC-143379
TCMDC-143191
